# Supplementary material for: Accelerometry as a method for external workload monitoring in invasion team sports. A systematic review
Source: PLoS One. 2020 Aug 25;15(8):e0236643. doi: 10.1371/journal.pone.0236643 (PMC7447012; doi:10.1371/journal.pone.0236643)
Supplement: S1 Table — (DOCX) [file pone.0236643.s001.docx]

| **S1 Table.** *Selected articles in goal striking games.* | | | | | | | | | | | |  |
| --- | --- | --- | --- | --- | --- | --- | --- | --- | --- | --- | --- | --- |
| Art. | Sport Context | Participants | | | Sport | Device  (Company)  Location | Accelerometer technical features | Accelerometry-based indexes | Unit of analysis | Results | Referential values | Quality  Index  (%) |
|  |  | Sex | Level | N |  |  |  |  |  |  |  |  |
|  |  |  |  |  |  |  |  |  |  |  |  |  |
| [83] | Training | ♂ | Junior  - U15  - U17  - U19 | 151 | Soccer | SPI-Pro X II (GPSport)  Scapulae | 3D-Accel  100 Hz  Reliable: No  Valid: No | Impacts Impacts/min  (>5 G) | 9 weeks with 4 90-minutes sessions where small-sided games were performed | Statistical differences were found in impacts between U-15 and the rest of categories. No differences were shown between U-17 and U-19. Besides, a great variability was found due to not realise a difference between playing positions. | Total impacts (>5 G)  U15 (490,8 ± 309.5)  U17 (584 ± 363.5)  U19(613.1 ± 329.4)  Impacts/min (>5 G)  U15 (8.2 ± 5.2)  U17 (9.7 ± 6.1)  U19 (10.2 ± 5.5) | 80.0% |
| [122] | Competition  Fatigue  Injury | ♂ | Professional  U21 | 573 | Soccer | MinimaxX S4 (Catapult Sports) Scapulae | 3D-Accel  100 Hz  Reliable: Yes  Valid: Yes | PL  PL(x)  PL(y)  PL(z)  Locomotor Efficiency | 574 matches  2 seasons  (12-13 and 13-14)  3 teams | PL and m/min were reduced from 15 minutes in all matches. The relationship of PL and m/min is higher in the last 15 minutes of each period. The PL variation between matches is explained by the individual and matches difference, but not by the playing position. The locomotor efficiency is reduced in the last minutes of each half and its related with fatigue and injury risk. |  | 85.7% |
| [123] | Competition | ♂ | Junior | 38 | Soccer | MinimaxX S3  (Catapult Sports) Scapulae | 3D-Accel  100 Hz  Valid: Yes  Reliable: Yes | PL | 8 official matches | According to playing position, significant differences was found. A performance decreasing between first and second half was shown, being these differences no significative. No differences were found in accelerations and decelerations at maximum intensity between first and second half. | PL all matches (Average)  Lateral defender:782.25±169.40  Central defender:745.84±161.40  Midfielder:991.49±223.23 Wingers:866.12±147.40 Forwards:892.33±209.21 | 73.3% |
| [47] | Training Competition | ♂ | Junior | 40 | Soccer | MinimaxX S3 (Catapult Sports) Scapulae | 3D-Accel  100 Hz  Reliable: Yes  Valid: No | PL/min  PL/min (x)  PL/min (y)  PL/min (z) | SSG (2vs2, 3vs3 y 4vs4) and 6 official matches | A reduction of number of players and playing area provoke higher neuromuscular load, being the most important load in vertical axis. According to playing positions, the midfielder presented the highest demands. | PL/min  Official match: 10.18 ± 2.12  2 vs 2: 15.00 ± 3.53  3 vs 3: 14.68 ± 3.27  4 vs 4: 13.47 ± 3.35 | 73.3% |
| [97] | Training  Injury | ♂ | Junior  Professional | 32 | Soccer | Viper V.2  (Stat Sports)  Scapulae | 3D-Accel  100 Hz  Reliable: No  Valid: No | Total Load | Relational analysis between injury risk and training load during two seasons. | A higher Total Load and accelerations in a period upper to 3-weeks is related to a higher injury risk without contact. A lower Total Load is related with injuries with contact. | Total Load/week  Low: 0-129  Low to moderate: 130-301  Moderate to high: 302-473  High: 474-647  Very high: >648 | 73.3% |
| [6] | Training | ♂ | Junior | 18 | Soccer | SPI-Pro  (GPS Sports)  Scapulae | 3D-Accel  100 Hz  Reliable: No  Valid: No | Force Load  Force Load/min | 3 Training Sessions  1. Strength  2. Speed  3. Endurance | Running-specific measures of neuromuscular function assessed in the field via GPS-embedded accelerometers show acceptable levels of reliability. Although the 3 sessions examined may be associated with limited neuromuscular fatigue, changes in neuromuscular performance and propulsion efficiency are likely session-objective dependent. | Force load and Force load/min  Strength: 49±7 / 0.6±0.1  Endurance: 47±11 / 0.5±0.1  Speed: 50±9 / 0.7±0.1 | 78.6% |
| [124] | Training | ♂ | Amateur | 10 | Soccer | JOHAN Sports (Noordwijk)  Scapulae | 3D-Accel  100 Hz  Valid: No  Reliable: No | PL | SSG 5vs5  with small goals  1. 3x6'/2'  2. 6x3'/2'  3. General training session | In the case of the 3x6’ regimen, large negative correlations between DOMS and TD (-0.68, [-0.89;-0.20]), JD (-0.66, [-0.89;-0.17]) and SD (-0.63, [-0.88;- 0.12]), and very large negative correlations between DOMS and PL (-0.84, [-0.95;-0.53]) were found. Very large (-0.73, [-0.91;-0.30] and large (-0.61, [-0.87;- 0.09]) negative correlations between DOMS and HRavg and PL, respectively, were observed during the 6x3’ regimen. | PTL  6X3': 20.79  3X6`: 38.26  General: 29.53 | 80.0% |
| [125] | Training | ♂ | Professional | 22 | Soccer | JOHAN Sports (Noordwijk)  Scapulae | 3D-Accel  100 Hz  Valid: No  Reliable: No | PL | First four weeks of the season | In the 10 vs 10 format, large increases in TD (27.9%, [17.7; 39.1]; ES: 3.54, [2.34; 4.74]) and PL (27.4%, [12.6; 44.1]; ES: 2.46, [1.20; 3.72]) were observed in the regular condition when compared to the ball possession condition. Between-formats analyses revealed that, in the 10 vs 10 format, when compared to the 5 vs 5 format, RD was very likely larger (123.5%, [33.7; 273.7]), as was SD (195.8%, [20.5; 626.2]). However, very likely large decreases in PL were observed in the 10 vs 10 format (-19.6%; [-29.4; -8.3]) in the ball possession condition. | PL  5x5 BP: 6.19±0.85  5x5 Regular: 6.30±1.01  10x10 BP: 5.50±0.43  10x10 Regular: 7.00±0.55 | 60.0% |
| [126] | Training  Competition | ♂ | Professional | 27 | Soccer | JOHAN Sports (Noordwijk)  Scapulae | 3D-Accel ±16g  100 Hz  Valid: No  Reliable: No | PL | All training sessions and matches during the 2018-2019 season | Correlations between the weekly training loads and the match demands of the same week were small for PL (r = 0.250 [0.13;0.36]), ACC (r = 0.292 [0.17;0.40]) and DEC (r = 0.236 [0.11;0.35]). This study reveals that ratios of above 1 were observed for specific measures (e.g., HSR, SD). It was also observed that training sessions are not adjusted according to weekly variations in match demands. |  | 66.7% |
| [26] | Competition | ♂ | Professional | 31 | Soccer | ZXY SportTracking  (Radionor Communications)  Center of mass | 3D-Accel  Valid: No  Reliable: Yes | PL/metros | 45 matches during 2009, 2010 and 2011 seasons (Rosenborg FC) | It is found statistical differences in PL/meters between playing positions, being highest in central defenders and lowest in lateral defenders. Therefore, it is necessary to monitoring the individualize demands to design training sessions. | PL/metros  Central defender: 1.31 ± 0.25 Lateral defender: 1.01 ± 0.13 Midfielder: 1.18 ± 0.14  Wingers: 1.20 ± 0.17  Forwards: 1.19 ± 0.09  All team: 1.17 ± 0.20 | 86.7% |
| [106] | Competition | ♂ | Elite | 23 | Soccer | Optimeye S5 (Catapult Sports) Scapulae | 3 3D-Accel  Reliable: No  Valid: No | PL  PL/min | 6 in-season weeks with 9 official matches | Our main findings showed significant contributions of the Player Load (r = .62, p < .001; 42.3%) and Anaerobic Threshold (r = − .199, p = .05, 17%) for the predictive model of Session-RPE. Physical measures and external load have a significant influence on the internal load in elite soccer players. Our findings suggest that sport scientists can use the Session-RPE as a low-cost method for prescribing and monitoring training loads. | PL: 826.7±189.07  PL/min: 9.88±1.18 | 66.7% |
| [127] | Training | ♂ | Junior | 41 | Soccer | WIMU PRO  (RealTrack Systems)  Scapulae | 4 3D-Accel  Reliable: No  Valid: No | PL_RT_  PL_RT_/min  Impacts | Training sessions during 3 months | The results indicated that the students who followed the TGA method recorded higher iTL values (heart rate) and spent more time performing high-intensity activities. Boys recorded higher eTL, iTL, and sRPE values than girls. There was an evolution in the sRPE between the assessments, with both groups presenting a more efficient sRPE in the posttest. The TGA method favors student physical fitness and health, thus, this method is recommended when planning physical education sessions. |  | 60.0% |
| [95] | Training | ♂ | Elite | 22 | Soccer | Viper v2 (StatSports)  Scapulae | 3D-Accel  100 Hz  Valid: No  Reliable: No | Impacts  Dynamic Stress Load  Impacts/min  DSL/min | 38 training weeks | Total high-speed-running (HSR; >14.4 km/h) distance and number of impacts and accelerations >3 m/s2 remained in the final multivariate model (P < .001). The adjusted correlations with sRPE were r = .14, r = .09, and r = .25 for HSR, impacts, and accelerations, respectively. For sRPE-TL, the correlations were r = .11, r = .45, and r = .37, respectively. Conclusions: The external-load measures that were found to be moderately predictive of sRPE-TL in soccer training were HSR distance and the number of impacts and accelerations. | DSL = 96±57  Impacts = 1898±730  DSL/min = 1.7±0.8  Imp/min = 63±8 | 73.3% |
| [51] | Training Competition | ♀ | University | 25 | Soccer | Zephyr Bioharness (Zephyr Technology)  Sternum | 3D-Accel  100 Hz  Valid: Yes  Reliable: Yes | Impulse Load | 17 official matches and 24 training sessions | Several very large to nearly perfect correlations were found between Impulse Load and total distance (r = 0.95; p< 0.001), Impulse Load and sRPE (r = 0.84; p < 0.001), and total distance and sRPE (r = 0.82; p < 0.001). | Impulse Load  Matches: 20120±8609  Training: 12410±4067 | 93.3% |
| [39] | Training  Competition | ♂ | Junior | 20 | Soccer | WIMU PRO  (RealTrack Systems)  Scapulae | 100 Hz  4 3-D Accel (±16, ±16, ±32, ±400g)  Valid: No  Reliable: No | PL_RT_/min  Impacts/min | One-month competitive period (4 training sessions with SSG and 4 official matches) | The SSGs presented walking and jogging intensity movements (0.7–7 to 7–14 km/h), with a 5-to-8 %HIA (high intensity activity, >16 km/h), where low intensity accelerations, decelerations and impacts were predominant (1–2.5 m/s2; 5–7 G). Only SSG4 presented similar demands to competition, finding differences between SSGs (p < 0.05; d = 1.40 ± 0.36). In conclusion, the objective of the SSGs directly influenced the demands on the players in training sessions. | PL_RT_/min / Impacts/min  SSG1: 1.47 ± 0.25 / 13.93 ± 8.32  SSG2: 1.21 ± 0.33 / 10.71 ± 7.28  SSG3: 1.06 ± 0.43 / 8.91 ± 8.21  SSG4: 1.34 ± 0.28 / 13.11 ± 8.42  Matches: 1.80 ± 2.95 / 10.46 ± 6.62 | 80.0% |
| [100] | Training | ♂ | Junior | 18 | Soccer | WIMU PRO  (RealTrack Systems)  Scapulae | 100 Hz  4 3-D Accel (±16, ±16, ±32, ±400g)  Valid: No  Reliable: Yes | PL  PL_RE_  PL_RT_  Total Load  Acel  Impulse Load | Three official matches | In absolute and centered data, very large to nearly perfect correlations (1st period: r > 0.803, p > 0.01; 2nd period: r > 0.919; p > 0.01) were found. Instead, very large differences were found in absolute values (bias = 579,226.6 to 285,931.1; t = 224.66 to 213.91, p < 0.01), and no differences in scaled and centered values (bias = 0; t = 1; p = 1). In conclusion, considering the different output (magnitude and units) among ABELIs, the standardization of a universal index to calculate accelerometer load is needed in order to make possible between-study comparison. | First period / Second period  PL: 579.85 / 467.2  PL_RE_: 556.51 / 473.7  PL_RT_: 58.17 / 46.88  Total Load: 57.98 / 46.72  Acel: 285989.29 / 241933.93  Impulse Load: 29162.65 / 24670.26 | 80.0% |
| [120] | Training | ♂ | Junior | 20 | Soccer | WIMU PRO  (RealTrack Systems)  Scapulae | 100 Hz  4 3-D Accel  Valid: Yes  Reliable: Yes | PL_RE_ | 23 training sessions | The main results indicate that exist an influence of subjective load variables in objective load variables (p<.05), except in simultaneous player percentage (p>.05), finding a high correlation between loads (r>.84). The task characteristics directly modify the load demands, being subjective tools as SIATE useful for their register when technological devices are not available. |  | 86.7% |
| [117] | Competition | ♂ | Professional | 37 | Soccer | Optimeye S4 (Catapult Sports) Scapulae | 100 Hz  3-D Accel  Valid: No  Reliable: Yes | PL/min PLx/min  PLy/min | 79 official matches during 2 seasons  (15-16 y 16-17) | The LMM did not identify any significant interactions or main effects for the PL/m, PLx/m, or PLy/m data, with average values for a 15 minute bout of match play being 0.139 ± 0.002 a.u/m, 0.036 ± 0.001 a.u/m, 0.035 ± 0.001 a.u/ m respectively. The LMM did however identify a significant main effect (p = 0.005) for time with the PLy/m data. With the exception of 30–45 minute period (0.068 ± 0.001 au/m), significantly higher PLy/m data was recorded in the 0–15 minute period of the matches (0.069 ± 0.001 au/m) when compared to all other time points (p ≤ 0.021; d < 0.09). Significantly lower PLVert/m was also recorded in the 75–90 minute period when compared to the 15–30 and 30–45 minute periods. | PL/min: 0.14±0.01 a.u./m  PLx/min: 0.04±0.01 a.u./m  PLy/min: 0.04±0.01 a.u./m | 86.7% |
| [104] | Competition | ♂ | Elite | 26 | Soccer | WIMU PRO  (RealTrack Systems)  Scapulae | 100 Hz  4 3-D Accel  Valid: No  Reliable: No | Acel | 42 official matches | Match location had an impact on HSRD (p<0.01; ES=0.05), DIS0-6 (p<0.01; ES = 0.05), and ACCMAX (p<0.01; ES= 0.05). Match outcome had a relation to TD (p<0.01; ES = 0.05), DIS0-6 (p<0.01; ES= 0.05) and HSRD (p<0.01; ES=0.05). Length of the microcycle had an impact on TD (p<0.01; ES=0.05), DIS0-6 (p<0.01; ES=0.11), ACC (p<0.01; ES= 0.04) and VMAX (p<0.01; ES=0.04). | Acel  Home win: 0.61±0.42  Home draw: 0.58±3.72  Home loss: 0.56±4.18  Away win: 0.60±3.23  Away draw: 0.61±4.10  Away loss: 0.58±1.71 | 80.0% |
| [88] | Competition | ♂ | Elite | 24 | Hockey | MinimaxX S4 (Catapult Sports) Scapulae | 100 Hz  3-D Accel  Valid: No  Reliable: Yes | PL  PL/min | 7 official matches and 7 training sessions | Significant differences between playing positions was found, being the lowest demands performed by the strikers and the highest by the defenders. | PL and PL/min  Strikers: 577±67/13.8±1.1  Attack midfield: 624±130/13.8±1.6  Defense midfield: 602±61/12.8±1.0  Defenders: 649±114/12.5±1.4  All Team: 617±106/13.2±1.4 | 86.7% |
| [118] | Training | ♂ | Professional | 11 | Soccer | Team AMS  (GPS Sports)  Scapulae |  | Body Load | Pre-post test  30-15 Intermittent Fitness Test,  5 training weeks | The players’ VIFT showed a most likely moderate improvement (+4.3%, 90% confidence limits [3.1; 5.5%], effect size ES, 0.70 [0.51; 0.89]). Accumulated NBL, Banister’s TRIMP and Edwards’ TRIMP showed large associations (r = 0.51 to 0.54) with changes in VIFT. Very large relationship was also observed between accumulated Z5 TRIMP (r= 0.72) with changes in VIFT. Large-to-nearly perfect within-individual relationships were observed between NBL and some of the other training metrics (i.e., Edwards’ TRIMP, Banister’s TRIMP, training duration, and total distance) in 10 out of 11 players. | Training load per session  Body Load= 126.3±30.1 | 80.0% |
| [128] | Competition | ♂ | Professional | 21 | Soccer | WIMU PRO  (RealTrack Systems)  Scapulae | 3D-Accel  100 Hz  Valid: Yes  Reliable: No | PL_RT_ | 12 official matches | Power Metabolic and PL_RT_ were measured. The main results indicated: (1) a performance reduction in both variables over the course of match time, (2) significant differences in both variables based on the specific position, (3) differences in physical demands during the season matches, (4) winning during a game period and the condition of being the visitor team provoked higher demands, and (5) a high correlation between both variables in soccer. | PL_RT_  First half: 64.74 ± 11.88  Second half: 54.30 ± 16.38 | 86.7% |
| [129] | Competition | ♀ | Elite | 30 | Soccer | MinimaxX S4 (Catapult Sports) Scapulae | 100 Hz  Reliable: No  Valid: No | PL/min | 10 official matches | Within match comparisons revealed that player load decreased significantly (p ≤ 0.05) in the second half (ES: 0.4). Despite generally small reductions in performance measures, there is evidence that accumulated fatigue throughout a multi-day tournament would affect performance negatively. | PL/min  Forwards: 8.3 ± 0.9  Midfielders: 9.7 ± 1.9  Defenders: 8.6 ± 1.6  First half: 9.1 ± 1.6  Second half: 8.5 ± 2.1 | 73.3% |
| [84] | Competition | ♀ | Elite | 45 | Soccer | MinimaxX S4 (Catapult Sports) Scapulae | 100 Hz  Reliable: No  Valid: No | PL  PL/min | 55 international official matches during 5 seasons | Total distance per minute exhibited the smallest variation (CV = 6.8–7.2%). Sprint-efforts were the most variable during a full-match (CV = 53%), whilst high-speed running per minute exhibited the greatest variation in the post-peak5-min period (CV = 143%). Variability of accelerations (CV = 17%) and PL (CV = 14%) was lower than that of high-speed actions. Positional differences were also present, with centre-backs exhibiting the greatest variation in high-speed movements (CV = 41–65%). | PL and PL/min  Centre-backs: 982±159/10.3±1.7  Lateral-backs: 1007±147/10.6±1.5  Midfielder: 1265±237/13.2±2.5  Forward: 1016±226/10.6±2.4  Team: 1096±239/11.5±2.5 | 86.7% |
| [119] | Competition | ♂ | Elite | 4 | Soccer | MinimaxX S4 (Catapult Sports) Scapulae | No technical features  Valid: Yes  Reliable: Yes | PL | 3 official matches during 7 days | Post-MD2, there were very or most likely harmful effects of extra-time on CMJ height (-6±9%), muscle soreness (+18±12%), and fatigue (+27±4%) scores, and overall wellness score (-13± 5%) compared to post-MD1. Furthermore, there were very likely harmful effects on muscle soreness (+13±14%), wellness scores (-8±10%), and CMJ height (-6±9%) post-MD3 vs. post-MD1. An extra-time match negatively impacted recovery 36 h post-match. Furthermore, in some players, index of performance in a 90 min match played 64h following extra-time were compromised, with subsequent recovery also adversely affected. | PL  Match 1: 10.3±1.4  Match 3: 10.9±2.7 | 93.3% |
| **Note.** ♂: Male; ♀: Female; CMJ: Counter-movement jump; Collisions: Total number of collisions; Collisions/min: collisions per minute; CV: Coefficient of variation; DOMS: Delayed Onset Muscle Soreness; Dynamic Stress Load: Accelerometer load index related to the weight of impacts; DSL/min: Dynamic Stress Load per minute; Force Load: accelerometer-load in 3 axes; Force Load/min: Force load per minute; HR: Heart rate; Impacts: total number of impacts; Impacts/min: Impacts per minute; Locomotor efficiency: Contribution of MD: Match Day; PL(y) in relation to total PL; PL: PlayerLoad^TM^ (sum of 3-axis); PL(x): PL^TM^ x-axis; PL(y): PL^TM^ y-axis; PL(z): PL^TM^ z-axis; PL/min: PL^TM^ (sum of 3-axis) per minute; PL(x)/min: PL^TM^ x-axis per minute; PL(y)/min: PL^TM^ y-axis per minute; PL(z)/min: PL^TM^ z-axis per minute; PL/meters: PL^TM^ (sum of 3-axis) per meter; PL2D: PL^TM^ in 2-axis; PL2D/min: PL^TM^ in 2-axis per minute; PLslow: PL^TM^ where travel speed is <2 m/s; PLslow/min: PL^TM^slow per minute; RD: Total distance covered per minute; sRPE: Session rated perceived exertion; SSG: Small-sided games; TD: Total distance covered; Total Load: Accelerometer load in 3-axis of movement; VO_2_max: Maximal oxygen consumption. | | | | | | | | | | | | |

**References**

6. Buchheit M, Lacome M, Cholley Y, Simpson BM. Neuromuscular Responses to Conditioned Soccer Sessions Assessed via GPS-Embedded Accelerometers: Insights Into Tactical Periodization. Int J Sports Physiol Perform. 2018;13:577–83.

26. Dalen T, Jørgen I, Gertjan E, Havard HG, Ulrik W. Player Load, Acceleration, and Deceleration During Forty-Five Competitive Matches of Elite Soccer. J Strength Cond Res. 2016;30:351–359.

39. Gómez-Carmona C, Gamonales J, Pino-Ortega J, Ibáñez S. Comparative Analysis of Load Profile between Small-Sided Games and Official Matches in Youth Soccer Players. Sports. 2018;6:173.

47. Beenham M, Barron DJ, Fry J, Hurst HH, Figueirdo A, Atkins S. A Comparison of GPS Workload Demands in Match Play and Small-Sided Games by the Positional Role in Youth Soccer. J Hum Kinet. 2017;22:129-137.

51. Gentles J, Coniglio C, Besemer M, Morgan J, Mahnken M. The Demands of a Women’s College Soccer Season. Sports. 2018;6:16.

83. Abade EA, Gonçalves BV, Leite NM, Sampaio JE. Time-Motion and Physiological Profile of Football Training Sessions Performed by Under-15, Under-17, and Under-19 Elite Portuguese Players. Int J Sports Physiol Perform. 2014;9:463–70.

84. Trewin J, Meylan C, Varley MC, Cronin J. The match-to-match variation of match-running in elite female soccer. J Sci Med Sport. 2018;21:196–201.

88. Polglaze T, Dawson B, Hiscock DJ, Peeling P. A Comparative Analysis of Accelerometer and Time–Motion Data in Elite Men’s Hockey Training and Competition. Int J Sports Physiol Perform. 2015;10:446–451.

95. Gaudino P, Iaia FM, Strudwick AJ, Hawkins RD, Alberti G, Atkinson G, et al. Factors Influencing Perception of Effort (Session Rating of Perceived Exertion) during Elite Soccer Training. Int J Sports Physiol Perform. 2015;10:860–864.

97. Bowen L, Gross AS, Gimpel M, Li F-X. Accumulated workloads and the acute:chronic workload ratio relate to injury risk in elite youth football players. Br J Sports Med. 2017;51:452–459.

100. Gómez-Carmona CD, Pino-Ortega J, Sánchez-Ureña B, Ibáñez SJ, Rojas-Valverde D. Accelerometry-Based External Load Indicators in Sport: Too Many Options, Same Practical Outcome? Int J Environ Res Public Health. 2019;16:5101.

104. Oliva-Lozano JM, Rojas-Valverde D, Gómez-Carmona CD, Fortes V, Pino-Ortega J. Impact Of Contextual Variables On The Representative External Load Profile Of Spanish Professional Soccer Match-Play: A Full Season Study. Eur J Sport Sci. 2020;Epub:Ahead of print.

106. Enes A, Oneda G, Alves DL, Palumbo D de P, Cruz R, Moiano Junior JVM, et al. Determinant Factors of the Match-Based Internal Load in Elite Soccer Players. Res Q Exerc Sport. 2020;Epub:Ahead of print.

117. Jones RN, Greig M, Mawéné Y, Barrow J, Page RM. The influence of short-term fixture congestion on position specific match running performance and external loading patterns in English professional soccer. J Sports Sci. 2019;37:1338–1346.

118. Rabbani A, Kargarfard M, Castagna C, Clemente FM, Twist C. Associations Between Selected Training Stress Measures and Fitness Changes in Male Soccer Players. Int J Sports Physiol Perform. 2019;14:1050-1057.

119. Winder N, Russell M, Naughton R, Harper L. The Impact of 120 Minutes of Match-Play on Recovery and Subsequent Match Performance: A Case Report in Professional Soccer Players. Sports. 2018;6:22.

120. Gómez-Carmona CD, Gamonales-Puerto JM, Feu S, Ibáñez SJ. Study of internal and external load by different instruments. A case study in grassroots. Sport Sci J Sch Sport Phys Educ Psychomot. 2019;5:444–468.

122. Barrett S, Midgley A, Reeves M, Joel T, Franklin E, Heyworth R, et al. The within-match patterns of locomotor efficiency during Professional Soccer match play: Implications for Injury risk? J Sci Med Sport. 2016;19:810–815.

123. Barron DJ, Atkins S, Edmundson C, Fewtrell D. Accelerometer derived load according to playing position in competitive youth soccer. Int J Perform Anal Sport. 2014;14:734–743.

124. Clemente FM. Associations between wellness and internal and external load variables in two intermittent small-sided soccer games. Physiol Behav. 2018;197:9–14.

125. Clemente FM, Praça GM, Bredt S da GT, Linden CMI van der, Serra-Olivares J. External Load Variations Between Medium- and Large-Sided Soccer Games: Ball Possession Games vs Regular Games with Small Goals. J Hum Kinet. 2019;70:191–198.

126. Clemente FM, Rabbani A, Conte D, Castillo D, Afonso J, Truman Clark CC, et al. Training/Match External Load Ratios in Professional Soccer Players: A Full-Season Study. Int J Environ Res Public Health. 2019;16:3057.

127. García-Ceberino JM, Antúnez A, Feu S, Ibáñez SJ. Quantification of Internal and External Load in School Football According to Gender and Teaching Methodology. Int J Environ Res Public Health. 2020;17:344.

128. Reche-Soto P, Cardona-Nieto D, Diaz-Suarez A, Bastida-Castillo A, Gomez-Carmona C, Garcia-Rubio J, et al. Player Load and Metabolic Power Dynamics as Load Quantifiers in Soccer. J Hum Kinet. 2019;69:259–269.

129. Strauss A, Sparks M, Pienaar C. The Use of GPS Analysis to Quantify the Internal and External Match Demands of Semi-Elite Level Female Soccer Players during a Tournament. J Sports Sci Med. 2019;11:73-81.
