# Supplementary material for: Accelerometry as a method for external workload monitoring in invasion team sports. A systematic review
Source: PLoS One. 2020 Aug 25;15(8):e0236643. doi: 10.1371/journal.pone.0236643 (PMC7447012; doi:10.1371/journal.pone.0236643)
Supplement: S2 Table — (DOCX) [file pone.0236643.s002.docx]

| **S2 Table.** *Selected articles in goal throwing games.* | | | | | | | | | | | | |  |
| --- | --- | --- | --- | --- | --- | --- | --- | --- | --- | --- | --- | --- | --- |
| Art. | Sport Context | | Participants | | | Sport | Device  (Company)  Location | Accelerometer technical features | Accelerometry-based indexes | Unit of analysis | Results | Referential values | Quality  Index  (%) |
|  |  |  | Sex | Level | N |  |  |  |  |  |  |  |  |
| [54] | Competition | | ♂ | Junior | 12 | Basketball | Optimeye S6 (Catapult Sports) Scapulae | 3D-Accel  100 Hz  Valid: Yes  Reliable: Yes | PL  PL/min | 8 official matches | Separate one-way ANOVAs revealed statistically significant differences between 1 min, 5 min, 10 min, and full game periods for Player Load, F (3,168) = 231.80, np2 = 0.76, large, p < 0.001. It is worth noting that guards produced a statistically significantly higher Player Load in 5 min (p < 0.01, np2 = 0.69, moderate), 10 min (p < 0.001, np2 = 0.90, moderate), and full game (p < 0.001, np2 = 0.96, moderate) periods than forwards. | PL / PL/min  1 min: 19.15±2.74 / 19.15±2.74  5 min: 63.79±9.55 / 12.76±1.91  10 min: 108.16±15.04 / 10.82±1.50  Full game: 370.67±105.85 / 7.53±1.53 | 93.3% |
| [24] | Competition | | ♀ | Elite | 12 | Netball | X8-mini (GulfCoast Data Concepts)  Centro de masas | 3D-Accel  100 Hz  Reliable: Yes  Valid: Yes | PL | 2 official matches | Off-ball guarding produced the highest player load per instance, while jogging produced the greatest player load per match. Non-locomotor activities contributed least to total match load for attacking positions (GS, GD and WA) and most for defending positions (GK, GD and WD). Specifically, C produced the greatest jogging load, WA and WD accumulated the greatest running load, while GS and WA accumulated the greatest shuffling load. WD and C accumulated the greatest guarding load, while WD and GK accumulated the greatest off-ball guarding load. | Per match PL Locomotor  Standing: 124.2±50  Walking: 256.1±69.7  Jogging: 376.8±239.1  Shuffling: 229.4±153.8  Running: 46.8±33.1  Per match PL Non-locomotor  Jump: 47.4±20.6  Off-ball guard: 303.5±208.8  Guard: 78.7±59.8  Defend: 75.6±38.7 | 92.9% |
| [130] | Competition | | ♀ | Elite | 11 | Netball | MinimaxX S5 (Catapult Sports)  Scapulae | 3D-Accel  100 Hz  Reliable: Yes  Valid: Yes | PL/min | 3 official matches | Following match 1, CK increased, whereas BAM+, JH, C and T decreased. Following two matches, BAM+, PPO, and T decreased with CK increasing versus baseline. Following consecutive matches, CK (likely moderate; 27.9% ± 19.5%) and C (possibly moderate; 43.3% ± 46.8%) increased, whilst BAM+ (possibly moderate; −20.6% ± 24.4%) decreased. Three days post-tournament BAM+, T, PPO, and JH decreased. Mid-court elicited higher mean HR (possibly moderate; 3.7% ± 3.8%), internal and external intensities (possibly very large; 85.7% ± 49.6%) compared with goal-based positions. | PL/min  Match 1: 7.9±1.9  Match 2: 8.2±2.3  Match 3: 8.7±2.6 | 86.7% |
| [131] | Competition | | ♀ | Elite | 12 | Netball | Optimeye S6 (Catapult Sports) Scapulae | 3D-Accel  100 Hz  Reliable: No  Valid: No | PL  PL/min  PL2D  PLslow  PLx  PLy  PLz | 6 official matches | Mean total distance-covered in match-play differed substantially between positions. Centre position accumulated the highest mean distance (5462.1 ± 169.4 m), whilst the Goal Shooter consistently covered the lowest mean distance (2134 ± 102.6 m). Change of direction relative to movement area was highest for the two most restricted positions based on average acceleration per 10 m covered during match-play (Goal Shooter; 7.21 ± 0.88 m · s−2 and Goal Keeper; 6.75 ± 0.37 m · s−2, remaining positions; 5.71 ± 0.14 m · s−2). | PL/min  Goal shooter: 6.38±0.34  Goal attack: 9.57±0.30  Wing attack: 10.11±0.51  Centre: 12.09±0.90  Wing defence: 9.36±0.82  Goal defence: 10.29±0.37  Goal Keeper: 7.31±0.56 | 71.4% |
| [49] | Training  Competition | | ♀ | University | 8 | Netball | MinimaxX S4 (Catapult Sports) Scapulae | 3D-Accel  100 Hz  Reliable: Yes  Valid: No | PL/min  PL/min (x)  PL/min (y)  PL/min (z) | 4 official matches and 15 training sessions | Centers presented the highest values in 3D PL and in PL of each axis (TE=0.67-0.91). The unique task that produced a similar competition demands was the training of specific competition skills. | PL/min  Match: 6.1  Skills: 6.0  Game-based: 9.0  Traditional: 18.5  HIIT: 16.6 | 86.7% |
| [85] | Competition | | ♀ | Elite | 32 | Netball | MinimaxX Team 2.5 (Catapult Sports) Scapulae | 3D-Accel  100 Hz  Valid: No  Reliable: Yes | PL/min PL(x)/min  PL(y)/min  PL(z)/min | 4 teams were registered during 5 official matches | A decrease in PL/min was found along the match in all playing roles. Centres presented the highest load while shooters presented the lowest load. | PL/min (average of all periods) Shooters: 9.4 ± 3.6 Centres: 10.6 ± 2.4 Defenders: 9.8 ± 2.7 | 80.0% |
| [105] | Competition | | ♂ | Amateur | 11 | Basketball | WIMU PRO  (RealTrack Systems)  Scapulae | 4 3D-Accel  100 Hz  Reliable: Yes  Valid: Yes | PL/min  Impacts/min | 6 official final round games | (a) guards covered more volume of displacements (effective on-court time: p < 0.01, E2R = 0.05; steps/min: p < 0.01, E2R = 0.28) and the centers performed competitive actions of higher load ([>8G]Imp/min: p < 0.01, E2 R = 0.20; jumps/min: p < 0.01, E2R = 0.33); (b) a performance decreasing was found between the first and second half of the game; (c) in balanced matches there was the most individual technical performance (PIR/min: p < 0.98, E2R = 0.01), while in the unbalanced games more  high-intensity impacts were seen ([>8G] Imp/min: p < 0.01, E2R = 0.07). | PL/min  Guards: 3.44±0.35  Forwards: 3.19«0.32  Centres: 3.24±0.29  Impacts/min (>3G)  Guards: 90.86±31.25  Forwards: 82.14±17.49  Centres: 88.35±12.69 | 93.3% |
| [48] | Training  Competition | | ♂ | Professional | 15 | Basketball | Optimeye S5 (Catapult Sports) Scapulae | 3D-Accel  100 Hz  Reliable: No  Valid: No | PL  PL/min | 9 weeks during competitive period (2-4 training sessions and 2 friendly games per week) | PL (arbitrary units [AU]) and EED (m) were statistically significantly (p < .05) higher during physical conditioning and games-based training respect to competition. Summated heart rate zones were statistically significantly (p < .05) higher during physical conditioning and games-based training than they were during competition. | PL  Physical conditioning: 632±139  Games-based training: 624±113  Competition: 449±118  PL/min  Physical conditioning: 6.5±0.8  Games-based training: 6.1±0.8  Competition: 4.35±1.1 | 80.0% |
| [132] | Competition | | ♂ | Professional | 5 | Basketball | Optimeye S5 (Catapult Sports) Scapulae | 3D-Accel  100 Hz  Reliable: Yes  Valid: No | PL  PL/min | 19 official games | PL, the number of absolute and relative jumps, high-intensity accelerations, absolute and relative total decelerations, total changes-of-direction, SHRZ, session-RPE, and RPE were higher during away games | PL / PL/min  Win: 579 ± 73 / 5.6 ± 0.7  Loss: 586 ± 73 / 5.9 ± 0.7  Home: 549 ± 73 / 5.6 ± 0.7  Away: 616 ± 73 / 6.0 ± 0.7 | 80.0% |
| [113] | Training  Competition | | ♂ | Professional | 8 | Basketball | Optimeye S5 (Catapult Sports) Scapulae | 3D-Accel  100 Hz  Reliable: Yes  Valid: Yes | PL  PL/min | One season (2018) 18 official games and 26 training sessions | Absolute PL, jumps, accelerations, decelerations, COD, and high-intensity jumps and accelerations were higher during 3-game than 1- and 2-game weeks (P <0.05, ES = 0.69–2.63). Absolute SHRZ and sRPE were higher during 3-game than 1- and 2-game weeks (P <0.05, ES = 0.86–2.43). Players completed similar individual game workloads regardless of the number of games played on consecutive days in the week. Workloads were similar during 1- and 2-game weeks, while the addition of a third game significantly increased the overall weekly workloads encountered. | PL / PL/min  Game 1: 541±187 / 5.34±1.77  Game 2: 575±166 / 5.71±1.71  Game 3: 529±221 / 5.39±2.26 | 93.3% |
| [133] | Competition | | ♂ | Referees | 9 | Basketball | WIMU PRO  (RealTrack Systems)  Scapulae | 4 3D-Accel  100 Hz  Reliable: Yes  Valid: Yes | PL_RT_  PL_RT_/min  Impacts | 15 games in an official championship | The first period was the period in which the greatest work demand was experienced in relation to these neuromuscular outcomes (11.92 PL; 3.61 Met; 277 Impacts). The results revealed a diminishment of internal and external demands on the referees over the course of the game. | PL_RT_: 44.13±7.92  PL_RT_/min: 0.56±0.08  Impacts: 1025±372 | 93.3% |
| [55] | Competition | | ♀ | Professional | 28 | Netball | Optimeye S5 (Catapult Sports) Scapulae | 3D-Accel  100 Hz  Reliable: Yes  Valid: No | PL/min | All official matches in one season | Across all time periods post 30-s, only one comparison was not meaningfully different i.e. three-thirds v two-thirds at the one-minute timepoint (effect size: 0.27, CL −0.05 to 0.60). Findings justify that netball athletes, depending on positional group defined by this study,should train at different intensities dependent on a specified duration. | PL/min  One third: 52.67±6.64  Two-third: 57.63±7.61  Three-third: 60.77±7.63 | 66.7% |
| [134] | Training | | ♂ | Universitary | 13 | Basketball | Optimeye S6 (Catapult Sports) Scapulae | 3D-Accel  100 Hz  Reliable: Yes  Valid: Yes | PL  PL/min  PL2D  PLx  PLy  PLz | 3 training sessions in off-season phase | Significant (p < 0.05) differences were observed in PL, PL2D, PL1D-FWD, PL1D-SIDE, PL1D-UP, and Distance across practices. Significant correlations (p < 0.001) existed between Distance and PL parameters (Practice 1: r = 0.799–0.891; Practice 2: r = 0.819–0.972; and Practice 3: 0.761–0.891). Predictive models using Distance travelled accounted for 73.5–89.7% of the variance in PL. Significant relationships and predictive capacities exists between systems. Nonetheless, each system also appears to capture unique information that may still be useful to performance practitioners regarding the understanding of eTL. | PL  Practice 1: 420.4 ± 102.9  Practice 2: 472.8 ± 109.5  Practice 3: 295.1 ± 57.8  PL/min  Practice 1: 5.8 ± 1.4  Practice 2: 5.1 ± 1.2  Practice 3: 5.3 ± 1.0 | 86.7% |
| [114] | Training | | ♂ | Universitary | 14 | Basketball | Optimeye S6 (Catapult Sports) Scapulae | 3D-Accel  100 Hz  Reliable: Yes  Valid: Yes | PL  PL/min  PL2D  PLx  PLy  PLz | 22 training sessions | PlayerLoad per minute was significantly higher during W1 and W2 (5.4 ± 1.3au and 5.3 ± 1.2au, respectively; p < 0.05) compared to subsequent weeks, but no additional differences in eTL parameters across time were observed. Scholarship  athletes displayed greater PlayerLoad; Inertial Movement Analysis (IMA), with no observed differences in eTL by position. | PL: 706.5  PL/min: 4.95  PL2D: 456.7  PLx: 285.2  PLy: 455.1  PLz: 296.3 | 80.0% |
| [135] | Competition | | ♀ | Junior | 34 | Netball | Optimeye S5 (Catapult Sports) Scapulae | 3D-Accel  100 Hz  Reliable: Yes  Valid: Yes | PL/min  PL-2D/min  PLx/min  PLy/min  PLz/min | 20 matches of a national championship | The O19 players recorded a higher mean distance  (3,365.7 6 1,875.1 m) per-match than U19 (p = 0.0095) players. The O19 players recorded a higher PL (p = 0.0003), PLx (p = 0.004), PLz (p = 0.0039), and PLVy(p = 0.0352) tan the domestic players. Domestic players recorded a higher maximal velocity than O19 players (p = 0.0003; d = 0.32) throughout the study. Domestic players recorded a higher average maximal heart rate (202.2 6 28.2 b /min) than O19 (p<0.0001) and U19 (p = 0.0002) players. | PL/min: 8.5±3.6  PL-2D/min: 5.6±3.6  PLx/min: 3.5±1.4  PLy/min: 5.9±2.6  PLz/min: 3.2±1.3 | 93.3% |
| [136] | Competition | | ♀ | Elite | 8 | Handball | Optimeye S5 (Catapult Sports) Scapulae | 3D-Accel  100 Hz  Reliable: Yes  Valid: No | PL  PL/min | 7 LMRL matches (30-min half) and 7 WBHL (20-min half) matches | The main outcomes revealed a PL.min–1 of ~9 AU and ~84% of HRmax per match. Moreover, significantly higher total PL (p<0.001; ES (effect  size) = Moderate) and match load calculated from S-RPE (p<0.05; ES= small to moderate) were found in LMRL compared to WBHL. Conversely, no statistically significant differences were found for PL.min–1 and %HRmax between the two leagues. | PL / PL/min  20-min half match time: 313.8 ± 110.3 / 5.0 ±1.4  30-min half match time: 418.3 ± 141.2 / 5.0 ±1.6 | 86.7% |
| [137] | Competition | | ♂ | Junior | 19 | Handball | WIMU PRO  (RealTrack Systems)  Scapulae | No technical features  Reliable: No  Valid: No | PL_RT_/min | One final tournament match | The result of the game is not a predictor of the physical performance of the team. No differences between teams was found in PL_RT._ | PL_RT_/min  Winner: 0.73  Losser: 0.76 | 66.7% |
| [50] | Training  Competition | | ♂ | Elite | 11 | Basketball | MinimaxX S3  (Catapult Sports) Scapulae | 3D-Accel  100 Hz  Reliable: No  Valid: No | PL/min | 3 official matches and different training sessions | No differences were found in physical and physiological variables between defensive and offensive actions. Accelerometric load (PL) was greater in official matches in relation to 5vs5 training situations. | PL/min  5vs5 during training sessions:  1.71 ± 0.84  Official matches:  2.79 ± 0.58 | 73.3% |
| [138] | Training | | ♂ | Professional | 13 | Basketball | Optimeye S5 (Catapult Sports) Scapulae | 3D-Accel  100 Hz  Reliable: Yes  Valid: No | PL/min | 12 training sessions | External workload was anticipated in 0–55% of future sessions using an error range of ±5%, and in 58–89% of sessions using an error range of ±10%. External workload during 5 vs. 5 games based drills can be anticipated in most sessions  using normative values established during an short-term monitoring period with an error range of ±10%. | PL/min: 6.43 | 73.3% |
| [42] | Training  Competition | | ♀ | University | 5 | Basketball | Optimeye S5 (Catapult Sports) Scapulae | 3D-Accel  100 Hz  Reliable: Yes  Valid: Yes | PL  IMA  PLy  PLz  PLx | 20 weeks during competition period (training and matches) | A great variability of loads along the 20 weeks of competition period was found. This variability influenced in the tensiomyographical activity of rectus femoris and biceps femoris. | 20 weeks average  PL= 4073 ± 900  IMA= 959 ± 228  PLy= 448±115  PLx= 291±86  PLz=218±66 | 93.3% |
| [27] | Competition | | ♂ | Junior | 94 | Basketball | WIMU PRO (RealTrack Systems)  Scapulae | 3D-Accel  100 Hz  Reliable: Yes  Valid: Yes | PL_RT_/min | 13 matches  4-days tournament (1 game per day) | According to team quality, the best teams had lower RD (p = 0.04; d = −0.14). Guards presented higher RD (p < 0.01; ω_p_^2^= 0.03), PSpeed (p < 0.01; ω_p_^2^= 0.01) and PAcc (p < 0.01; ω_p_^2^= 0.02) compared to forwards and centers. The first quarter showed differences with higher RD (p < 0.01; ω_p_^2^= 0.03), %HIR (p < 0.01; ω_p_^2^= 0.02), and PL (p < 0.01; ω_p_^2^= 0.04) compared to all other quarters. The third match of the tournament presented higher demands in RD (p < 0.01; ω_p_^2^= 0.03), HIR (p < 0.01; ω_p_^2^= 0.01) and PL (p < 0.01; ω_p_^2^= 0.02) compared with the first two matches. | PL/min  Guard: 1.4±0.3  Forward: 1.3±0.3  Center: 1.3±0.4 | 100% |
| [89] | Competition | | ♂ | Professional | 15 | Lacrosse | MinimaxX S4 (Catapult Sports) Scapulae | 3D-Accel  100 Hz  Valid: No  Reliable: Yes | PL/min | 4 national official matches | A performance decreasing is produced in all playing positions along the match. Besides, in PL/min index, the midfielder presented the highest demands while the defenders presented the lowest demands. These data indicate the important of individualize training loads to performance enhancement. | PL/min  Midfielders: 9.9 ± 1.5 Forwards: 8.2 ± 2.1 Defenders: 7.6 ± 2.7 | 80.0% |
| [139] | Competition | | ♂ | Junior | 25 | Basketball | WIMU PRO (RealTrack Systems)  Scapulae | 3D-Accel  100 Hz  Valid: No  Reliable: No | PL_RT_  PL_RT_/min | 3 official matches in a 4-days tournament |  | PL_RT_  Guards: 45±31  Forwards: 47±30  Centers: 37±20  PL_RT_/min  Guards: 0.90±0.40  Forwards: 0.97±0.37  Centers: 1.00±0.35 | 66.7% |
| [28] | Training  Competition | | ♀ | Amateur | 10 | Basketball | WIMU PRO (RealTrack Systems)  Scapulae | Valid: Yes  Reliable: Yes | PL_RT_/min  Impacts/min | Last 2 months of the season. 22 training sessions and 8 official matches | The results obtained showed that the load experienced during competition was significantly higher (p < 0.001) than during training (Heart Rate, Player Load, Steps, Jumps, and Impacts). There were also differences according to playing positions, mainly between the backcourt and frontcourt players (p < 0.001). | PL_RT_/min / Impacts/min  Training: 0.94 / 1.69  Competition: 2.82 / 1.65 | 86.7% |
| [8] | Training | | ♂ | Professional | 8 | Basketball | MMA7361L (Freescale Semiconductor)  Scapulae | 4 3D-Accel  100 Hz  Valid: Yes  Reliable: Yes | PL | 44 training sessions | Significant moderate relationships were observed between PL and sRPE (r=0.49, 95% CI=0.23–0.69, p=0.001) and TRIMP models (r=0.38, 95% CI=0.09–0.61, p=0.011). A significant large correlation was evident between external training load and the SHRZ model (r=0.61, 95%CI=0.38–0.77, p<0.001). Although significant relationships were found between internal and external training load models, the magnitude of the correlations and low commonality suggest that internal training load models measure different constructs of the training process than the accelerometer training load model in basketball settings. |  | 86.7% |
| [29] | Training | | ♂ | Elite | 12 | Basketball | X8-mini (GulfCoast Data Concepts)  Center of mass | 3D-Accel  100 Hz  Valid: Yes  Reliable: Yes | PL | Different SSG (2x2, 3x3, 4x4 y 5x5) both on half and full court. | On full court, 3x3 and 5x5 presented the greatest accelerometer demands. Instead, on half court, 2x2 presented the highest demands. | PL Full Court  2x2: 14.6 ± 2.8 3x3: 18.7 ± 4.1 4x4: 13.8 ± 2.5 5x5: 17.9 ± 4.6  PL Half Court  2x2: 12.7 ± 2.7 3x3: 10.9 ± 1.8 4x4: 10.8 ± 2.3 5x5: 12.0 ± 5.6 | 93.3% |
| [115] | Training | | ♀ | Elite | 9 | Basketball | ActiLife v12 (Actigraph)  Scapulae | 3D-Accel  100 Hz  Reliable: No  Valid: Yes | AcelT | 18 official matches | Back-court players experienced more supramaximal bouts (125 ± 37 vs. 52 ± 36; p = 0.031) of greater average duration (2.1 ± 0.4 vs. 1.4 ± 0.2 s; p = 0.021) and maximum duration (7 ± 2 vs. 3 ± 1 s; p = 0.020). More sedentary to very light activity was observed in the 2nd and 4th quarters compared to the 1st and 3rd quarters (p < 0.05). Despite reduced playing time, bench players performed similar amounts of maximal and supramaximal exercise when compared to starters (p ≥ 0.279). |  | 66.7% |
| [43] | Training  Competition | | ♂ | Elite | 13 | Basketball | Optimeye S5 (Catapult Sports) Scapulae | 3D-Accel  100 Hz  Reliable: No  Valid: Yes | PL  PLx  PLy  PLz | 5 months competitive period (5-10 training sessions and 2-3 matches per week) | A significant correlation was observed between the external load variables and sRPE (range r=0.71–0.93). Additionally, the sRPE variable showed a high correlation with the total PL, ACC, DEC, and CoD. A stronger correlation was found between PL and total ACC, DEC and CoD. The only contrary finding was the correlation between PL and JUMP variables, which showed a stronger correlation for hJUMP. Tri-axial accelerometry technology and the sRPE method serve as valuable tools for monitoring the training load in basketball. | Sessions average  PL: 314.9±90  PLx: 132±37.3  PLy: 127.4±37.4  PLz: 2016.1±59.9 | 86.7% |
| [140] | Competition | | ♀ | Junior | 11 | Netball | SPI-PRO  (GPSport)  Scapulae | No technical features  Valid: No  Reliable: No | Body Load  Impacts | 8 competitive matches | The main findings were that players travelled less  distance during a match than previously reported. The Centre covered significantly greater distances than the other positions (p<0.001). The Goal Shooter and Goal Keeper covered the least distance and endured lower accelerometer loads than other positions (p<0.001). Very low levels of very high speed running and sprint efforts were observed across all positions. | Body Load  Goal shooter: 29744  Goal attack: 152377  Wing attack: 83600  Centre: 141871  Wing defence: 92516  Goal defence: 89143  Goal Keeper: 41962 | 66.7% |
| [116] | Competition | | ♂ | Elite | 12 | Basketball | Viper V.2  (Statsports)  Scapulae | 3D-Accel  100 Hz  Valid: No  Reliable: Yes | Total Load | 2 tournament matches in 2-days | Power Forwards had the lowest external load (Point guards = Small forwards > Shooting guards > Centers > Power Forwards) | Point guards (4.8±1.1) Small forwards (4.8±0.8) Shooting guards (4.6±1.7) Centers (4.4±0.3) Power Forwards (3.5±1.1) | 86.7% |
| [141] | Competition | | ♂ | Junior | 94 | Basketball | WIMU PRO (RealTrack Systems)  Scapulae | 3D-Accel  100 Hz  Reliable: No  Valid: No | PL_RT_ | 13 official games | Results from the locomotor ratio at both lower and higher speeds presented a significant effect for the quarter (F = 7.3, p < 0.001 and F = 7.1, p < 0.001, respectively) and player position (F = 3.1, p = 0.04, F = 9.2, p < 0.001, respectively). There was an increase in the locomotor ratio from game quarter (Q) Q1 to Q4 at lower speeds, but contrary trends at higher speeds, i.e., the values have decreased from Q1 to Q4. Also, forwards and centers of the best teams presented lower values at higher speeds. |  | 66.7% |
| [62] | Competition | | ♂ | Elite | 6 | Handball | Optimeye S5 (Catapult Sports) Scapulae | 3D-Accel  100 Hz  Reliable: Yes  Valid: No | PL/min | 9 official matches | Individual profiles in relation to playing positions were found. The neuromuscular load is reduced along the match, except in the last 5-minutes of each half where the accelerometer load is increased. | PL/min  Outfield players: 9.52 ± 1.1  Backs: 9.76 ± 1.4  Wings: 9.18 ± 0.6  Pivots: 9.31 ± 0.8 | 85.7% |
| [25] | Training  Competition | | ♀ | Elite | 12 | Netball | X8-mini (GulfCoast Data Concepts)  Centro de masas | 3D-Accel  100 Hz  Reliable: Yes  Valid: Yes | PL  PL/min | 15 official matches and 17 training sessions | Player load in matches for the goal-based positions (GS, GK & GD) tended to be lower than the attacking and wing-based positions (GA, WA, WD & C). The difference was largely due to the amount of time spent in the low intensity activity. Playing intensity of matches was greater than training sessions, however the total time spent in moderate to high intensity activities was not practically different. | PL  Training Sessions: 177.6 Matches: 154.7 | 80.0% |
| **Note.** ♂: Male; ♀: Female; Acc: Positive changes of speed; Acel: Accelerometer load; CMJ: Counter-movement jump; CoD: Changes of direction; Collisions: Total number of collisions; Collisions/min: collisions per minute; CV: Coefficient of variation; Dec: Negative changes of speed; HR: Heart rate; IMA: Index for accelerative events; Locomotor efficiency: Contribution of MD: Match Day; PL(y) in relation to total PL; PL: PlayerLoad^TM^ (sum of 3-axis); PL(x): PL^TM^ x-axis; PL(y): PL^TM^ y-axis; PL(z): PL^TM^ z-axis; PL/min: PL^TM^ (sum of 3-axis) per minute; PL(x)/min: PL^TM^ x-axis per minute; PL(y)/min: PL^TM^ y-axis per minute; PL(z)/min: PL^TM^ z-axis per minute; PL/meters: PL^TM^ (sum of 3-axis) per meter; PL2D: PL^TM^ in 2-axis; PL2D/min: PL^TM^ in 2-axis per minute; PLslow: PL^TM^ where travel speed is <2 m/s; PLslow/min: PL^TM^slow per minute; RD: Total distance covered per minute; sRPE: Session rated perceived exertion; SSG: Small-sided games; TD: Total distance covered; Total Load: Accelerometer load in 3-axis of movement; VO_2_max: Maximal oxygen consumption. | | | | | | | | | | | | | |
|  | |  | | | | | | | | | | | |

**References**

8. Scanlan AT, Wen N, Tucker PS, Dalbo VJ. The relationships between internal and external training load models during basketball training. J Strength Cond Res. 2014;28:2397–2405.

24. Bailey JA, Gastin PB, Mackey L, Dwyer DB. The Player Load Associated with Typical Activities in Elite Netball. Int J Sports Physiol Perform. 2017;12:1218-1223.

25. Young CM, Gastin PB, Sanders N, Mackey L, Dwyer DB. Player Load in Elite Netball: Match, Training, and Positional Comparisons. Int J Sports Physiol Perform. 2016;11:1074–1079.

27. Pino-Ortega J, Rojas-Valverde D, Gómez-Carmona CD, Bastida-Castillo A, Hernández-Belmonte A, García-Rubio J, et al. Impact of Contextual Factors on External Load During a Congested-Fixture Tournament in Elite U’18 Basketball Players. Front Psychol. 2019;10:1100.

28. Reina M, García-Rubio J, Feu S, Ibáñez SJ. Training and competition load monitoring and analysis of women’s amateur basketball by playing position: approach study. Front Psychol. 2018;9:2689.

29. Schelling X, Torres L. Accelerometer Load Profiles for Basketball-Specific Drills in Elite Players. J Sports Sci Med. 2016;15:585–591.

42. Peterson KD, Quiggle GT. Tensiomyographical responses to accelerometer loads in female collegiate basketball players. J Sports Sci. 2017;35:2334–2341.

43. Svilar L, Castellano J, Jukic I. Load monitoring system in top-level basketball team: relationship between external and internal training load. Kinesiology. 2018;50:25–33.

48. Fox JL, Stanton R, Scanlan AT. A Comparison of Training and Competition Demands in Semiprofessional Male Basketball Players. Res Q Exerc Sport. 2018;89:103–111.

49. Chandler PT, Pinder SJ, Curran JD, Gabbett TJ. Physical demands of training and competition in collegiate netball players. J Strength Cond Res. 2014;28:2732–2737.

50. Montgomery PG, Pyne DB, Minahan CL. The Physical and Physiological Demands of Basketball Training and Competition. Int J Sports Physiol Perform. 2010;5:75–86.

54. Alonso E, Miranda N, Zhang S, Sosa C, Trapero J, Lorenzo J, et al. Peak Match Demands in Young Basketball Players: Approach and Applications. Int J Environ Res Public Health. 2020;17:2256.

55. Graham S, Zois J, Aughey R, Duthie G. The peak player load^TM^ of state-level netball matches. J Sci Med Sport. 2020;23:189–193.

62. Wik EH, Luteberget LS, Spencer M. Activity Profiles in International Women’s Team Handball Using PlayerLoad. Int J Sports Physiol Perform. 2017;12:934–942.

85. Cormack SJ, Smith RL, Mooney MM, Young WB, O’Brien BJ. Accelerometer Load as a Measure of Activity Profile in Different Standards of Netball Match Play. Int J Sports Physiol Perform. 2014;9:283–291.

89. Polley CS, Cormack SJ, Gabbett TJ, Polglaze T. Activity Profile of High-Level Australian Lacrosse Players: J Strength Cond Res. 2015;29:126–136.

105. Fernández-Leo A, Gómez-Carmona CD, García-Rubio J, Ibáñez SJ. Influence of Contextual Variables on Physical and Technical Performance in Male Amateur Basketball: A Case Study. Int J Environ Res Public Health. 2020;17:1193.

113. Fox J, O’Grady C, Scanlan AT. Game schedule congestion affects weekly workloads but not individual game demands in semi-professional basketball. Biol Sport. 2020;37:59–67.

114. Heishman A, Miller RM, Freitas EDS, Brown BS, Daub BD, Bemben MG. Monitoring External Training Loads and Neuromuscular Performance For Division I Basketball Players Over the Pre-Season. J Sports Sci Med. 2020;19:204-212.

115. Staunton C, Wundersitz D, Gordon B, Kingsley M. Accelerometry-Derived Relative Exercise Intensities in Elite Women’s Basketball. Int J Sports Med. 2018;39:822–827.

116. Vázquez-Guerrero J, Suarez-Arrones L, Casamichana Gómez D, Rodas G. Comparing external total load, acceleration and deceleration outputs in elite basketball players across positions during match play. Kinesiology. 2018;50:228–234.

130. Birdsey LP, Weston M, Russell M, Johnston M, Cook CJ, Kilduff LP. Neuromuscular, physiological and perceptual responses to an elite netball tournament. J Sports Sci. 2019;37:2169-2174.

131. Brooks ER, Benson AC, Fox AS, Bruce LM. Physical movement demands of elite-level netball match-play as measured by an indoor positioning system. J Sports Sci. 2020;Epub:ahead of print.

132. Fox JL, Stanton R, Sargent C, O’Grady CJ, Scanlan AT. The Impact of Contextual Factors on Game Demands in Starting, Semiprofessional, Male Basketball Players. Int J Sports Physiol Perform. 2020;15:450–456.

133. García-Santos D, Pino-Ortega J, García-Rubio J, Vaquera A, Ibáñez SJ. Internal and External Demands in Basketball Referees during the U-16 European Women’s Championship. Int J Environ Res Public Health. 2019;16:3421.

134. Heishman A, Peak K, Miller R, Brown B, Daub B, Freitas E, et al. Associations Between Two Athlete Monitoring Systems Used to Quantify External Training Loads in Basketball Players. Sports. 2020;8:33.

135. King DA, Cummins C, Hume PA, Clark TN. Physical Demands of Amateur Domestic and Representative Netball in One Season in New Zealand Assessed Using Heart Rate and Movement Analysis. J Strength Cond Res. 2018;Epub:ahead of print.

136. Kniubaite A, Skarbalius A, Clemente FM, Conte D. Quantification of external and internal match loads in elite female team handball. Biol Sport. 2019;36:311–316.

137. Mancha-Triguero D, Reina M, Baquero B, García-Rubio J, Ibáñez SJ. Analysis of the competitive load in u16 handballers as a function of the final result. E-Balonmano Com Rev Cienc Deporte. 2018;14:99–108.

138. O’Grady CJ, Dalbo VJ, Teramoto M, Fox JL, Scanlan AT. External Workload Can Be Anticipated During 5 vs. 5 Games-Based Drills in Basketball Players: An Exploratory Study. Int J Environ Res Public Health. 2020;17:2103.

139. Portes R, Jiménez SL, Navarro RM, Scanlan AT, Gómez M-Á. Comparing the External Loads Encountered during Competition between Elite, Junior Male and Female Basketball Players. Int J Environ Res Public Health. 2020;17:1456.

140. van Gogh MJ, Wallace LK, Coutts AJ. Positional Demands and Physical Activity Profiles of Netball: J Strength Cond Res. 2020;34:1422–1430.

141. Vázquez-Guerrero J, Fernández-Valdés B, Gonçalves B, Sampaio JE. Changes in Locomotor Ratio During Basketball Game Quarters From Elite Under-18 Teams. Front Psychol. 2019;10:2163.
