# Supplementary material for: Accelerometry as a method for external workload monitoring in invasion team sports. A systematic review
Source: PLoS One. 2020 Aug 25;15(8):e0236643. doi: 10.1371/journal.pone.0236643 (PMC7447012; doi:10.1371/journal.pone.0236643)
Supplement: S3 Table — (DOCX) [file pone.0236643.s003.docx]

| **S3 Table.** *Selected articles in try scoring games.* | | | | | | | | | | | |  |
| --- | --- | --- | --- | --- | --- | --- | --- | --- | --- | --- | --- | --- |
| Art. | Sport Context | Participants | | | Sport | Device  (Company)  Location | Accelerometer technical features | Accelerometry-based indexes | Unit of analysis | Results | Referential values | Quality  Index  (%) |
|  |  | Sex | Nivel | N |  |  |  |  |  |  |  |  |
| [142] | Competition | ♂ | Universitary | 43 | American football | Optimeye S5 (Catapult Sports)  Scapulae | 100 Hz  3D-Accel  Valid: No  Reliable: No | IMA | One season | Results indicated that DBs travelled significantly (p,0.05) greater distances than OL and WR, but not DL. For MV, DBs and WRs were not significantly different but were significantly different from OL and DL. Also, DL was significantly different than OL. For the most intense acceleration (3–10 m·s22), WR accelerated significantly further than all other positions and DBs accelerated further than DL and OL. There was not significant difference between DL and OL. For deceleration at the high-intensity measure, significant differences existed among all positions. Underestimation of workload during games could be a factor for the overuse and soft-tissue injuries and more serious injuries. |  | 60.0% |
| [44] | Training  Competition | ♂ | Elite  Professional | 40 | Australian football | MinimaxX S4 (Catapult Sports) Scapulae | 3D-Accel  100 Hz  Reliable: Yes  Valid: No | PL  PLslow | 24 elite-level official matches (AFL), 29 subelite-level official matches (Victorian League) and 32 training sessions. | Midfielders obtained a higher PL and PLslow both in elite and subelite respect to the rest of playing positions. The lowest values were found in deeps. Higher values were found in elite-level. The small-sided games were the specific task that obtained a higher correlation with competition demands. | PL/min midfielders (16.03 ± 4.21)  nomadics (14.96 ± 2.35)  deeps (11.01 ± 2.63) ruckmen (14.91 ± 3.30) PLslow/min  midfielders (4.19 ± 1.33)  nomadics (4.21 ± 0.90)  deeps (3.75 ± 0.90)  ruckmen (4.41 ± 0.78) | 86.7% |
| [143] | Training  Competition  Injuries | ♂ | Professional | 133 | Australian football | Optimeye S5 (Catapult Sports)  Scapulae | 100 Hz  3D-Accel  Valid: Yes  Reliable: No | PL | 3 seasons | Injury prediction models built using training load data from a single club showed poor ability to predict injuries when tested on previously unseen data, suggesting limited application as a daily decision tool for practitioners. |  | 93.3% |
| [144] | Competition | ♂ | Elite | 18 | Australian football | MinimaxX S5 (Catapult Sports) Scapulae | 100 Hz  3D-Accel  Valid: No  Reliable: No | PL | 16 official matches (AFL) | A variation of neuromuscular demands during 16 official matches was found. | PL (all team players)  Minimum: 1275.93 ± 36.38  Maximum: 1377.48 ± 31.47 | 73.3% |
| [145] | Competition | ♂ | Elite | 11 | Australian football | MinimaxX S5 (Catapult Sports) Scapulae | 3D-Accel  100 Hz  Valid: No  Reliable: No | PL | 3 official matches (AFL) | A variation of accelerometric load in relation to competition demands was shown. These variations affected directly the immunological function of the organism. | PL  M1: 1096 ± 115.1  M2: 1082 ± 90.4  M3: 1266 ± 124.6 | 73.3% |
| [32] | Training  Competition  Injuries | ♂ | Elite | 46 | Australian football | SPI-Pro X II (GPSport) Scapulae | 3D-Accel  100 Hz  Valid: No  Reliable: No | Force Load | 18 official matches and preseason matches and 2 weekly training sessions. | A strong relationship was found between the load accumulation and the increase of injury risk. No differences was found in the prevalence to suffer an injury between both categories. | Force Load  Pre-season: 27.613 (23.322-31.904) Season: 28.135 (22.423-32.567) | 66.7% |
| [92] | Training  Competition  Fatigue | ♂ | Elite | 17 | Australian football | MinimaxX Team 2.5 (Catapult Sports) Scapulae | 3D-Accel  100 Hz  Reliable: Yes  Valid: No | PL(x)/min  PL(y)/min  PL(z)/min | Comparative analysis between Yo-Yo IR2 and the perceived demands in competition. | Fatigue reduced the vertical vector contribution and also it had influence in aerobic and anaerobic capacity during Yo-Yo IR2 test performance. | % of change in PL/min x-axis: -0.4 ± 5.6 y-axis: 4.9 ± 6.2 z-axis: -5.1 ± 4.7 | 86.7% |
| [57] | Training | ♂ | Elite | 33 | Rugby | High Performance Unit (GPSports)  Scapulae | 3D-Accel  100 Hz  Reliable: Yes  Valid: No | Body Load  Body Load 2D | 8 preseason weeks (4-5 training sessions per week) | This was matched by a concomitant increase in distance (small), high speed running (small), very-high speed running (moderate) and two-dimensional (2D) BodyLoad (small) demands in positional skills. Hit-up forwards (674±253AU) , experienced greater 2D BodyLoad demands than outside backs (432±230 AU, p=.034). Within positional drills hit-up forwards experienced greater relative 2D BodyLoad demands than outside backs (p=0.015). |  | 80.0% |
| [146] | Training  Competition | ♂ | Professional | 48 | Rugby | Optimeye S5 (Catapult Sports)  Scapulae | 3D-Accel  100 Hz  Reliable: Yes  Valid: No | PL | One season daily data in pre-season and in-season | Injury-risk increased alongside increases in the ACWR for duration, total distance and PlayerLoad. Conversely, injury-risk decreased (Area Under Curve: 0.569–0.585) with increases in the four-weekly duration, total distance, accelerations, decelerations and PlayerLoad. For relative distance, high fourweekly workloads (high: >60 m min−1) demonstrated a positive association with injury-risk, whilst high two-weekly loads (high: >82 m min−1) were negatively associated. |  | 66.7% |
| [96] | Competition | ♂ | Elite | 2 | Rugby Union | SPI-Elite  (GPSport)  Scapulae | 3D-Accel  100 Hz  Reliable: No  Valid: No | Impacts  Body Load  Body Load/min | One competitive team selection game | Positional data revealed that the back performed a greater number of sprints (20 km/h) than the forward (34 vs. 19) during the game. Conversely, the forward entered the lower speed zone (6-12 km/h) on a greater number of occasions than the back (315 vs. 229) but spent less time standing and walking (66.5 vs. 77.8%). Players were found to perform 87 moderate-intensity runs (14km/h) covering an average distance of 19.7 m (SD = 14.6). Average distances of 15.3 m (back) and 17.3 m (forward) were recorded for each sprint burst (20 km/h), respectively. | Back / Forward  Impacts (>5G): 798 / 1274  Impacts (>10G): 4 / 13  Body Load: 31402 / 119103  Body Load/min: 376 / 1426 | 73.3% |
| [147] | Training | ♂ | Elite | 14 | Australian football | MinimaxX S3  (Catapult Sports)  Scapulae | 3D-Accel  100 Hz  Reliable: Yes  Valid: No | PL  PL2D | Comparative between 4 SSG with different modified situations | The specific characteristics of SSG modified the kinematical and neuromuscular demands. | PL  SSG1: 25.2 ± 2.6  SSG2: 27.5 ± 3.0  SSG3: 28.6 ± 2.9  SSG4: 25.8 ± 3.4 | 73.3% |
| [148] | Training | ♂ | Elite | 55 | Australian football | Optimeye S5 (Catapult Sports)  Scapulae | No technical features  Reliable: No  Valid: No | PL | 2 competitive season | High cumulative loads and acute-to-chronic workload ratios were associated with increased risk of injuries. The effects for measures derived using exponentially weighted moving averages were greater than those for rolling averages. History of a recent injury, long-term experience at professional level, and substantial reductions in a selection of musculoskeletal screening and subjective wellness scores were associated with increased risk. The effects of high cumulative loads were underestimated by ∼ 20% before adjusting for previous injuries, whereas the effects of high acute-to-chronic workload ratios were overestimated by 10–15%. Injury-prone players, identified via player identity in the mixed model, were at > 5 times higher risk of injuries compared to robust players (hazard ratio 5.4, 90% confidence limits 3.6–12) despite adjusting for training load and previous injuries. Combinations of multiple risk factors were associated with extremely large increases in risk; for example, a hazard ratio of 22 (9.7–52) was observed for the combination of high acute load, recent history of a leg injury, and a substantial reduction in the adductor squeeze test score. | 7-d rolling average  Pre-season  High: >350  Moderate-high: 280-350  Moredate-low: 200-279  Low: <200  In-season  High: >310  Moderate-high: 260-310  Moredate-low: 200-259  Low: <200 | 86.7% |
| [149] | Training | ♂ | Professional | 40 | Australian football | Optimeye S4 (Catapult Sports) Scapulae | 3D- Accel  Valid: No  Reliable: No | PL | Three week block at the end of the preseason phase of training | The ‘large’ SSG generated a greater absolute distance, relative distance, maximum velocity, PlayerLoad® and distance >4.16 m.s-1 compared to the ‘small’ and ‘medium’ SSGs. These results provide AF coaches with insights into how task constraint manipulation impacts the technical and physical profiles of players during small-sided game-play. | PL  Small: 19.5 ± 0.4  Medium: 20.8 ± 0.4  Large: 22.1 ± 0.4 | 73.3% |
| [45] | Training  Competition  Injuries | ♂ | Elite | 30 | Rugby | MinimaxX S3 (Catapult Sports) Scapulae | 3D-Accel  100 Hz  Valid: Yes  Reliable: No | Total collisions Collisions/min | 21 training sessions and 1 friendly match | According to playing position, statistical differences was found in number of collisions, being the hit-up forwards who had the highest demands and the outside backs who had the lowest demands. | Total collisions  Hit-up forwards: 37  Wide running forwards:28 Adjustables: 30 Outside backs: 16  Collisions/minute Hit-up forwards: 1.02 Wide running forwards: 0.59  Adjustables: 0.45 Outside backs: 0.48 | 80.0% |
| [46] | Training  Competition | ♂ | Elite | 30 | Rugby | MinimaxX Team 2.5 (Catapult Sports) Scapulae | 3D-Accel  100 Hz  Valid: Yes  Reliable: No | Total collisions Collisions/min | 124 training sessions and 16 official matches | The most collisions were performed by hit-up forwards and wide-running forwards. | Total collisions  Hit-up forwards: 42  Wide running forwards:45  Adjustables: 34 Outside backs: 28  Collisions/minute Hit-up forwards: 1.09 Wide running forwards:0.76  Adjustables: 0.58 Outside backs: 0.38 | 83.3% |
| [79] | Competition | ♂ | Professional | 182 | Rugby | MinimaxX S4 (Catapult Sports) Scapulae | 3D-Accel  100 Hz  Valid: No  Reliable: Yes | PL/min  PL2D/min PLslow/min Collisions/min | 26 official matches | PL obtained high correlations between number of collisions, total distance, low-intensity activity and repeated high-intensity efforts in forwards and hookers. Thus, it is useful to analyse this variable for monitoring competition demands. Instead, a low correlation was found in adjustables and outside backs. | PL/min  Forwards: 9.6 ± 2.0 Hookers: 10.4 ± 1.1 Adjustables: 8.7 ± 1.3 Outside backs: 7.2 ± 0.8 Colisiones/minuto Forwards: 0.77 ± 0.19 Hookers: 0.64 ± 0.16 Adjustables: 0.35 ± 0.10 Outside backs: 0.27 ± 0.10 | 86.7% |
| [80] | Competition | ♂ | Professional | 32 | Rugby | MinimaxX S4  (Catapult Sports) Scapulae | 3D-Accel  100 Hz  Valid: Yes  Reliable: No | Total collisions Collisions/min | An official match and different tests of strength, vertical jump and Yo-Yo IR1 | The strongest players performed greater total distance and high-intensity activity. Therefore, lower body strength is important to maximize performance. Besides, a higher total collisions and collisions/minute in forwards respect to backs. | Total collisions (+10G) Back: 26 ± 5 Forward: 32 ± 8 Collisions/minute (+10G) Back: 0.3 ± 0.1 Forward: 0.7 ± 0.2 | 86.7% |
| [150] | Training | ♂ | Elite | 36 | Australian football | MinimaxX Team 2.5 (Catapult Sports) Scapulae | 3D-Accel  100 Hz  Valid: No  Reliable: Yes | PL/min PLslow/min | 15 training sessions | The players that perceived a poor physical status before the training sessions, obtained lower performance in the detected variables by the inertial devices during the training sessions. | Training sessions average  PL/min: 7.7 ± 1.3  PLlento/min: 1.9 ± 0.3 | 80.0% |
| [93] | Training | ♂ | Elite | 42 | Australian football | MinimaxX Team 2.5 (Catapult Sports) Scapulae | 3D-Accel  100 Hz  Valid: No  Reliable: Yes | PL/min PLslow/min | 14 training sessions to compare the inertial device variables with sRPE. | The experience, the playing position and the tasks duration modified the relationship between the external load and sRPE. Thus, it is important to register both variables to avoid injury risk in athletes. | Training sessions  PL: 433 ± 133  PLslow: 114 ± 34 | 86.7% |
| [151] | Competition | ♂ | Elite | 20 | Australian football | MinimaxX S4 (Catapult Sports) Scapulae |  | PL | 4 official matches to determine the kinematical demands of the specific skills and to validate the impacts detection automatically by the accelerometer. | The device presented a high accuracy to detect high and moderate impacts, but not to detect low impacts. The skills with generated higher PL with contact were tackle and marking contest and without contact picked-up ball/bent over. | PL  Skills with contact  Mean value: 3.53 ± 1.36  Tackle: 3.92 ± 1.41 Wrestling: 2.69 ± 1.24  Skill without contact Mean value: 2.79 ± 1.16  Change of direction: 2.64 ± 0.97  Picked-up ball/bent over: 3.08 ± 1.25 | 86.7% |
| [152] | Training  Fatigue | ♂ | Professional | 23 | Australian football | MinimaxX Team 2.5 (Catapult Sports) Scapulae | 3D-Accel  100 Hz  Reliable: Yes  Valid: No | PL  PL/min  PLx  PLy  PLz | 1 competition week with evaluations: (a) baseline, (b) 48-hrs after, (c) 96-hrs after | Results: A small decrease in CMJH; ES -0.43 ± 0.39 (likely) was observed 48 hours post-match before returning to baseline 96 hours post-match. This was accompanied by corresponding moderate decreases in the SRT variables; PL1Dup; ES -0.60 ± 0.51 (likely) and PL1Dside; ES -0.74 ± 0.57 (likely) 48 hours post-match before also returning to pre-match baseline. Conclusion: The results suggest that in the presence of NMF, players utilise an alternative running profile to produce the same external output (i.e. time). This supports changes in accelerometer variables during a SRT can be used as an alternate method of measuring NMF in high performance ARF and provides a flexible option for monitoring changes within the recovery phase post-match. | Professional  PL=1295.7±116.3; PL/min=13.3±1.4  Semi-professional  PL=1172.3±138.9  PL/min= 12.9±1.4 | 80.0% |
| [60] | Training | ♂ | Elite | 12 | Rugby 7 | MinimaxX S4 (Catapult Sports) Scapulae | No technical features data  Reliable: No  Valid: No | PL | 3 training weeks to analyse the relationship between accelerometer load and CMJ test performance. | No significant differences were found between weeks in PL variable and test CMJ. Thus, a correct loads planning produced a correct performance keeping in the last matches of the season. | Training PL (mean of players)  Week 1: 323 ± 61  Week 2: 325 ± 64  Week 3: 312 ± 67 | 73.3% |
| [153] | Competition | ♀ | Elite | 20 | Rugby 7 | MinimaxX S4 (Catapult Sports)  Scapulae | No technical features data  Reliable: Yes  Valid: No | PL | 5 World Rugby Women’s Sevens Series events (2013-3014 season) | Total running distance, moderate-speed (0.2-3.5 m·s-1) and high-speed running (3.5-5.0 m·s-1) distances were significantly greater in the first half (20.1±4.1%, 17.6±6.9%, 24.5±7.8%). Time spent between 90-100% of maximum heart rate (16.4±14.5%) and Player LoadTM (19.0±5.1%) were significantly greater in the second half. No significant differences in physiological or activity profiles were observed between forwards and backs. |  | 80.0% |
| [41] | Training  Competition | ♂ | Professional | 45 | Australian football | Optimeye S5 (Catapult Sports) Scapulae | 3D-Accel  100 Hz  Reliable: Yes  Valid: No | PL | 24 weeks of a season analysing official matches and training sessions | Dose-response models from multiple training load inputs can predict within-individual variation of MEI.min-1 and PRscore. Internal and external training input methods exhibited comparable predictive power. | PL weekly  2513.5 ± 231.5 | 80.0% |
| [154] | Training  Injuries | ♂ | University | 58 | American football | Optimeye S5 (Catapult Sports) Scapulae | 3D-Accel  100 Hz  Reliable: No  Valid: No | PL | 8 weeks during competitive period (3 training sessions per week) | A one unit increase in wellness Z score and energy were associated with a trivial 2.3% (90% confidence interval (CI): 0.5, 4.2; SMD: 0.12) and 2.6% (90% CI: 0.1, 5.2; SMD: 0.13) increase in player load. A one unit increase in muscle soreness (players felt less sore) corresponded to a trivial 4.4% (90% CI: -8.4, -0.3; SMD: -0.05) decrease in s-RPE training load. |  | 80.0% |
| [155] | Training | ♂ | University | 16 | Rugby | Optimeye S5 (Catapult Sports) Scapulae | 3D-Accel  100 Hz  Reliable: No  Valid: No | PL | 3 sets of 6 collisions | High-speed running distance (r = 0.50, 95%CI -0.66-0.84) was related to EEVO2, while Player Load was not (r = 0.37, 95%CI -0.81-0.68). Whilst metabolic power might provide a different measure of external load than other typically used micro-technology metrics (e.g. high-speed running, Player Load), it underestimates energy expenditure during intermittent team sports that involve collisions. | PL: 54.4±5.5 | 78.6% |
| [156] | Competition | ♂ | Professional | 12 | Rugby | MinimaxX S4 (Catapult Sports)  Scapulae | 100 Hz  Reliable: No  Valid: No | PL  PL/min | 5 matches in two days | The low- and high-fitness groups reported similar mean HR, PlayerloadTM/min, and distance/min for matches. Further, the high-fitness group reported higher measures of tournament workload, including distance (ES = 0.71), PlayerloadTM (ES = 0.85) and  Edwards’ training impulse (TRIMP) (ES = 1.23). | PL  Average 5-matches: 240.2 ± 24.3  PL/min  Average 5-matches: 12.1 ± 1.22 | 66.7% |
| [110] | Competition | ♂ | Professional | 21 | Rugby | Optimeye S4 (Catapult Sports) Scapulae | 3D-Accel  100 Hz  Valid: Yes  Reliable: Yes | Collision | 2 competitive fixtures | High Yo-Yo and squat performance resulted in greater loads during match-play (p < 0.05). Post-match fatigue is lower in players with well-developed high-intensity running ability, and lower body strength, despite these players having greater internal and external match loads. |  | 86.7% |
| [58] | Training  Competition | ♂ | Professional | 44 | Australian football | Optimeye S5 (Catapult Sports) Scapulae | 3D-Accel  100 Hz  Reliable: Yes  Valid: No | PL | 2 pre-seasons periods of 21 weeks and 2 in-season periods of 23 weeks | Players in the high training load (HTL) group performed more relative total and high-speed distance in matches compared with the moderate (MTL; ES = 0.73–0.86) and low (LTL; ES = 0.68–1.31) training load groups, with the differences becoming greater as the season progressed. There were no clear differences in Player Rank score between groups. There were positive relationships between preseason high-speed running and match relative distance (p = 0.001; r = 0.417; r2 = 0.174) and match relative high-speed running (p = 0.001; r = 0.561; r2 = 0.314), which were greatest in the HTL group. | Pre-season loads  High load: 32981 ± 4063  Moderate load: 28251 ± 6010  Low load: 20105 ± 5292 | 80.0% |
| [53] | Competition | ♂ | Professional | 38 | Australian football | Optimeye S5 (Catapult Sports) Scapulae | 3D-Accel  100 Hz  Valid: No  Reliable: No | PL/min | 22 games of the 2017 AF League season | There were substantial reductions in average speed across each duration as the number of technical involvements increased, other than for the 10-minute period. The reductions in speed were greatest during the 1-minute period for 1 (ES 5 20.59 6 0.13), 2 (ES 5 21.96 6 0.17), and 3 (ES 5 22.39 6 0.27) involvements. Similarly, less pronounced reductions were seen for accelerometer load, other than during the 7- and 10-minute periods where there were small to moderate increases in load for periods with technical involvements. | PL/min : 12.3±5.2 | 60.0% |
| [157] | Competition | ♂ | Professional | 24 | Rugby | APEX  (StatSports)  Scapulae | 3D-Accel  100 Hz  Valid: No  Reliable: No | Total Load/min | 26 NRL matches and 22 ISC matches | Both professional and semi-professional forwards showed small to moderately higher accelerometer load compared to backs, which increased with period duration (ES range: 0.22–0.79). Similarly, acceleration demands were greater for forwards compared to backs across both playing standards, with moderate to large differences (ES range: 0.52–0.96). | Total Load/min  NRL forwards: 3.26 ± 0.47  ISC forwards: 3.31 ± 0.38  NRL backs: 3.16 ± 0.42  ISC backs: 3.25 ± 0.47 | 60.0% |
| [158] | Competition | ♂ | Professional | 33 | Rugby Union | MinimaxX S4 (Catapult Sports)  Scapulae | 3D-Accel  100 Hz  Valid: Yes  Reliable: No | PL/min  Collisions | 13 official matches | Significant positional differences were found for movement characteristics during performance (P < 0.05). Temporal analysis of all players displayed significant differences in player load, cruising and striding between halves, with measures of low- and high-intensity movement and acceleration/deceleration significantly declining throughout each half. | PL/min / Collisions  First Half: 6.9 ± 0.8 / 12.3 ± 9.5  Second Half: 6.5 ± 0.9 / 12.6 ± 9.8 | 80.0% |
| [159] | Competition | ♂ | Elite | 33 | Australian football | Team Sport 2.5 (Catapult Sports) Scapulae | No technical features  Valid: No  Reliable: Yes | PL | 31 official matches | While global measures of physical performance are relatively stable, higher-speed activities and technical measures exhibit a large degree of between-match variability in Australian Football. However, these measures remain relatively stable between positions, and within and between Australian Football League seasons. | PL  Start of the season: 1263  Middle-season: 1266  End of the season: 1246 | 85.7% |
| [160] | Training | ♂ | Elite | 117 | American football | Optimeye S5 (Catapult Sports) Scapulae | 3D-Accel  100 Hz  Reliable: Yes  Valid: Yes | PL | 73 training sessions in 2014-2015 season and 56 training sessions in 2015-2016 | Injuries were associated with greater increases in workload during the week of injury over the prior month when compared with uninjured controls. Injured players saw a 111% (95% CI 66%-156%) increase in workload whereas uninjured players saw a 73% (95% CI 34%-112%) increase in workload during the week of injury (P = 0.032). Individuals who had an acute to chronic workload ratio higher than 1.6 were 1.5 times more likely to sustain an injury relative to time- and position-matched controls (64.6% vs 43.1%, P = 0.004). |  | 60.0% |
| [161] | Competition | ♂ | Professional | 28 | Rugby Union | MinimaxX S4 (Catapult Sports)  Scapulae | 3D-Accel  100 Hz  Reliable: Yes  Valid: No | PL  PLslow | 15 official matches of 2012/2013 season | We found large between-match (within-player) variation for HSR (27.6%; ±90% confidence 41 limits 6.9% [forwards], 20.1%; ±4.1% [backs]), VHSR (68%; ±19%, 34.1%; ±7.5%), TI (24.0%; 42 ±5.9%, 36.4%; ±7.9%) and RHIE (18.7%; ±4.4%, 39.5%; ±8.8%), with moderate variability for match 43 RPE (8.2%; ±1.8%, 10.8%; ±2.1%), PL (7.3%; ±1.7%, 10.0%; ±2.0%) and PLSLOW (8.9%; ±2.0%, 44 10.7%; ±2.1%). Threshold values for likely substantial between-match changes in high-intensity 45 physical performance measures ranged from 21–76%, and ~10% for match RPE, PL and PLSLOW. | PL  All: 549±81  Forwards: 585±51  Backs: 521±89  PLslow  All: 251±45  Forwards: 286±31  Backs: 225±35 | 80.0% |
| [81] | Competition | ♂ | Elite | 22 | Rugby | SPI-Pro X II (GPSport) Scapulae | 3D-Accel  Reliable: No  Valid: No | Impacts | 8 official matches | Greater impacts were performed by the forwards with statistical differences in all intensities impacts. A relation between the neuromuscular response after and before the match with the number of collisions was shown. An increase of high-intensity collisions provoked greater fatigue after the match. | Total impacts  Forward  5-6 G: 260 ±43  6-6.5 G: 173±98  6.5-7 G: 212 ± 101  7-8 G: 154 ± 44  8-10 G: 55 ± 17  +10 G: 41 ± 22  Backs  5-6 G: 205 ± 106  6-6.5 G: 183 ± 65  6.5-7 G: 178 ± 49  7-8 G: 120 ± 57  8-10 G: 48 ± 36  +10 G: 32 ± 5 | 73.3% |
| [90] | Competition  Fatigue | ♂ | Elite | 15 | Australian football | MinimaxX Team 2.5 (Catapult Sports) Scapulae | 3D-Accel  100 Hz  Reliable: Yes  Valid: No | PL/min | 22 official matches and performance relation with Yo-Yo IR2 | A performance decreasing in PL/min along the match was found, especially in Q2 and Q4. Besides, a moderate correlation between the Yo-Yo IR2 values and PL/min performed in official matches was shown. | PL/min (media todos los jugadores) Q1: 16.3 (15.5-17.1) Q2: 15.3 (14.5-16.8)  Q3: 15.9 (15.1-16.8) Q4: 15.1 (14.2-15.8) | 86.7% |
| [162] | Training  Lesiones | ♂ | Junior | 63 | American football | Optimeye S5 (Catapult Sports) Scapulae | 3D-Accel  100 Hz  Reliable: No  Valid: No | PL | A regular season | An increase in acute (7d) load saw an increased stride variability in these athletes. Feeling less fatigued and/or lower muscle soreness was associated with higher stride variability. Conclusions: The assessment of variability has the potential to identify athletes who are displaying phys- ical symptoms that would indicate the need to modify training. | Acute Player Load (7-day mean) = 418.81±112.34 | 80.0% |
| [163] | Competition | ♂ | Elite | 33 | Rugby | SPI-HPU  (GPSport)  Scapulae | 3D-Accel  100 Hz  Reliable: Yes  Valid: Yes | Impacts  Body Load | All matches occurred over a 14-week period | Forwards sustained more (d = 0.44) high-intensity impacts and greater (d = 0.26) aggregated body demands. | Impacts (>7G) / Body Load  Forwards: 120 ± 55 / 106 ± 29  Backs: 99 ± 44 / 98 ±32  Front row: 125± 78 / 105 ± 37  Second row: 93 ± 26 / 92 ± 16  Back row: 138 ± 32 / 118 ± 23  Scrum half: 96 ± 19 / 109 ± 21  Inside backs: 93 ± 53 / 95 ±39  Outside backs: 106 ± 40 / 97 ± 27 | 86.7% |
| [164] | Training | ♂ | Junior | 170 | Rugby Union | Optimeye S5 (Catapult Sports) Scapulae | 3D-Accel  Reliable: No  Valid: No | PL  PL/min | 1 training week of 10 different teams | In relation to category and age, higher values provoked more physical and kinematical demands during training sessions. | PL and PL/min U16School: 262±41 and 5.3±1.0 U18School: 270±42 and 5.0±1.0 U16Club: 354±74 and 5.5±0.8 U18Club: 371±75 and 5.3±0.9 U16Academy: 316±53 and 6.5±0.8 U18Academy: 424±56 and 6.9±0.9 | 85.7% |
| [165] | Training | ♂ | Junior | 20 | Rugby Union | Optimeye S5 (Catapult Sports) Scapulae | 3D-Accel  100 Hz  Reliable: Yes  Valid: No | PL  PLslow | 10-week in-season period | Mean weekly sRPE was 1217 ± 364 AU (between-subject coefficient of variation (CV) = 30%), with a total distance (TD) of 11629 ± 3445 m (CV= 30%), and PlayerLoadTM (PL) of 1124 ± 330 AU (CV=29%). Within-subject CV ranged between 5-78% for sRPE, 24-82% for TD, and 19-84% for PL. Mean TD (13063 ± 3933 vs. 10195 ± 2242 m), and PL (1246 ± 345 vs. 1002 ± 279 AU) were both likely greater for backs compared to forwards (moderate ES), however differences in sRPE were unclear (small ES). | PL: 1124±330  PLslow: 542±165 | 73.3% |
| [112] | Competition  Training | ♂ | Junior | 61 | Rugby Union | Optimeye S5 (Catapult Sports) Scapulae | 3D-Accel  Reliable: Yes  Valid: Yes | PL  PL/min | 8 official matches and 15 training sessions | Schoolboy forwards were underprepared for low-intensity activities experienced during match-play, with schoolboy backs underprepared for all movement demands. Academy forwards were exposed to similar physical demands in training to matches, with academy backs similar to or exceeding values for all measured variables. | PL / PL/min  Schoolf forwards: 345±43 / 6.4±2.4  School backs: 399±141 / 6.5±1.3  Academy forwards: 350±48 / 5.8±0.5  Academy backs: 378±86 / 407±89 | 78.6% |
| [87] | Competition | ♂ | Junior | 112 | Rugby Union | Optimeye S5 (Catapult Sports) Scapulae | 3D-Accel  100 Hz  Reliable: Yes  Valid: Yes | PL/min PLslow/min | 6 official matches | The backs performed higher meters/min and high-intensity travel distance than the forwards. A higher category provoked higher demands. Instead, forwards performed higher PL/min and PLslow/min than backs. | PL/min  U16 Back: 6.7 U16 Forward: 7.4 U18 Back: 7.3 U18 Forward: 7.6  U20 Back: 6.4 U20 Forward: 6.9 | 100.0% |
| [166] | Competition | ♂ | Junior | 66 | Rugby Union | Optimeye S5 (Catapult Sports) Scapulae | 3D-Accel  100 Hz  Reliable: Yes  Valid: No | PLslow | 6 official matches | Academy forwards and backs almost certainly and very likely covered greater total distance than school forwards and backs. Academy players from both positions were also very likely to cover greater jogging distances. Academy backs were very likely to accumulate greater PLslow and the academy forwards a likely greater sprinting distance than school players in their respective positions. The MSS, total, walking and sprinting distances were greater in backs (likely-almost certainly), while forwards accumulated greater PLslow (almost certainly) and jogging distance (very likely). |  | 80.0% |
| [52] | Training  Competition | ♂ | Elite | 44 | Australian football | MinimaxX S4 (Catapult Sports) Scapulae | 3D-Accel  100 Hz  Reliable: Yes  Valid: Yes | PL | 41 weeks of competitive period, including training sessions and official matches. | During in-season, half the total load came from games and the remaining half from training, predominantly skills and  upper-body weights. Total distance, high-intensity running, and PL showed large to very large reductions from preseason to in-season, whereas changes in mean speed were trivial across all blocks. | PL  Training sessions  Pre-season: 1985±745  In-season: 1014 ± 383  Official matches Pre-season: 1010±290  In-season: 1320 ± 195 | 93.3% |
| [94] | Competition | ♂ | Professional | 26 | Rugby Union | Optimeye S5 (Catapult Sports) Scapulae | 100 Hz  3D-Accel  Valid: No  Reliable: Yes | PL  PL2D  PLslow Collisions | 6 official matches | All PL variables demonstrated very large relationships with collisions in the forwards, while PLslow demonstrated the largest relationship (large) with collisions in the backs. PLslow may provide the most useful metric for measuring collision-based activity in both positional groups during match-play. Additionally, nearly perfect and very large relationships were observed between PL and total distance for forwards and backs respectively, suggesting that PL can be successfully used to quantify running demands | PL  Forwards: 483 ± 113 Backs: 479 ± 102 PLslow  Forwards: 219 ± 52 Backs: 190 ± 41 Total collisions Forwards: 26 ± 9 Backs: 14 ± 6 | 80.0% |
| [167] | Training | ♂ | Professional | 20 | Rugby Union | Optimeye S5 (Catapult Sports) Scapulae | 100 Hz  Reliable: No  Valid: Yes | PLslow | Two weeks of a pre-season period | The inclusion of contact during field-based training almost certainly increased mean heart rate (9.7; ±3.9%) and sRPE (42; ±29.2%) and resulted in likely and very likely greater decreases in upper-body NMF (−7.3; ±4.7% versus 2.7; ±5.9%) and perception of well-being (−8.0; ±4.8% versus −3.4; ±2.2%) 24 h post-training, respectively, and almost certainly greater elevations in [CK] (88.2; ±40.7% versus 3.7; ±8%). The exclusion of contact from field-based training almost certainly increased running intensity (19.8; ±5%) and distance (27.5; ±5.3%), resulting in possibly greater decreases in lower-body NMF (−5.6; ±5.2% versus 2.3; ±2.4%) | PLslow  CON: 165 ± 1.17  nCON: 108 ± 1.13 | 80.0% |
| [111] | Training | ♂ | Elite | 21 | Soccer | Optimeye S5 (Catapult Sports) Scapulae | 3D-Accel  100 Hz  Valid: Yes  Reliable: Yes | PL/min  PL/m/min  PLslow/min  PL-2D/min  PL_AP_ (%)  PL_ML_ (%)  PL_V_ (%) | 110 training sessions  36 matches  **Prior Match**  1. 2x3 min 5vs5+5+gk SSG  2. CMJ  **Weekly volumen**  GPS, PL^TM^ and sRPE | All weekly load metrics increased SSG PL^TM^/min when above season average, however, the impact on FT:CT was trivial. Reduced weekly FT:CT compared to baseline resulted in lower SSG PL^TM^/min and PLslow/min. FT:CT below baseline increased match PL_ML_(%) and decreased PL_V_ (%) during subsequent match play. Similarly, a reduction in SSG PL/min was followed by increased match PL_ML_(%). | PL  HW: 2402±365  LW: 856±299  SSG 5vs5+5+gk  PL/min  HW (14.1±1.4); LW (14.6±1.5)  PL/m/min  HW (0.11±0.01); LW (0.12±0.01)  PL-2D/min  HW (8.57±0.87); LW (8.92±0.94)  PLslow/min:  HW (4.24±0.55); LW (856±299).  PL_AP_  HW (26.5±2%); LW (26.4±1.5%)  PL_ML_  HW (26.9±1.7%); LW (26.5±1.6%)  PL_V_  HW (47.5±2%); LW (47.3±2%) | 93.3% |
| [168] | Competition | ♂ | Elite | 18 | Soccer | Optimeye S5 (Catapult Sports) Scapulae | 3D-Accel  100 Hz  Reliable: Yes  Valid: Yes | PL | 3 pre-season matches | Measures of CMJ performance and hormonal concentrations were sensitive to levels of A League football match load. Although jump height was reduced immediately post-match, FT:CT provided a more sensitive measure of recovery. Football match play induces an acute hormonal response with substantial individual variability thereafter. | PL load volume:  Low=0-499  Medium=499-1000  High>1000 | 73.3% |
| [82] | Competition | ♂ | Amateur | 7 | Rugby Union | SPI Elite (GPSport) Scapulae | 3D-Accel  100 Hz  Reliable: No  Valid: No | Impacts | 3 official matches | The rugby union presented greater demands than rugby-11 and the training load should adapt. Forwards and backs performed different demands. Forwards suffer more impacts than backs. | Total impacts  Forward  5-6 G: 501.6 ± 106  6-6.5 G: 341.3 ± 219  6.5-7 G: 161.6 ± 107  7-8 G: 143.1 ± 122  8-10 G: 66.6 ± 48  +10 G: 10.4 ± 5  Backs  5-6 G: 382 ± 129  6-6.5 G: 326 ± 173  6.5-7 G: 54.3 ± 28.9  7-8 G: 29.8 ± 9  8-10 G: 35.2 ± 26  +10 G: 6.3 ± 4 | 66.7% |
| [169] | Competition | ♂ | Professional | 40 | Australian football | Team Sport 2.5 (Catapult Sports) Scapulae | No technical features  Valid: No  Reliable: Yes | PL | 15 official matches | Higher demands were found when the team was losing in kinematical variables (%HIA, sprints), but these differences were not found in neuromuscular variables. | PL/min  Win: 11.9  Loss: 12.0 | 86.7% |
| [170] | Competition | ♂ | Elite | 19 | Rugby Union | SPI-PRO  (GPSport)  Scapulae | 3D-Accel  100 Hz  Reliable: Yes  Valid: No | Impacts/min | 23 official matches in 2013 season | Forwards and backs adopt different pacing strategies regardless of bout type, with forwards demonstrating progressively greater performance decrements over the course of the match. These findings reflect differing physical demands, notably contact and running  loads, of players in different positions. | Impacts/min (>5G)  Forward / Backs  Q1: 11±5 / 10±4  Q2: 8±2 / 10±4  Q3: 8±4 / 10±3  Q4: 8±3 / 9±3 | 73.3% |
| [171] | Training  Competition | ♂ | Professional | 15 | Rugby | Viper Pod 2  (STATSports)  Scapulae | 3D-Accel  Reliable: No  Valid: No | PL | 9 training sessions and 4 official matches | Players experience a gradual decrease in neuromuscular function across an intensified competitive period. | PL  M1: 384  M2: 473  M3: 373  M4: 391 | 80.0% |
| [172] | Training | ♂ | Elite | 63 | American football | MinimaxX S4 (Catapult Sports) Scapulae | 3D-Accel  100 Hz  Reliable: Yes  Valid: No | PL  PL/min | 11 training sessions classified respect to match day (MD-4, MD-3, MD-2) | Moderate to large differences were observed between these two positions and the other positional groups. A similar relationship was observed in Player Load and Player Load per Minute, with the DB and WR groups performing greater amounts of load compared to other positional groups. |  | 80.0% |
| [173] | Training | ♂ | Professional | 17 | Rugby | SPI-PRO XII  (GPSport)  Scapulae | 3D-Accel  100 Hz  Reliable: No  Valid: No | Impacts  Body Load | Two 12-week preseason periods | Small-sided games and conditioning extracted 1 principal component, explaining 68% and 52% of the variance, respectively. Skills, wrestle, strongman, and speed extracted 2 principal components each explaining 68%, 71%, 72%, and 67% of the variance, respectively | Body Load / Impacts  Small-sided games: 79±85/ 1835±1819  Skills: 36±33 / 1069±965  Conditioning: 93±73 / 3202±2490  Speed: 28±18 / 603±400  Strongman: 9±13/ 391±428  Wrestle: 11±9 / 269±261 | 80.0% |
| [174] | Training | ♂ | Professional | 23 | Rugby | Optimeye S4 (Catapult Sports) Scapulae | 3D-Accel  100 Hz  Reliable: Yes  Valid: Yes | PL | All training sessions in pre-season period (12 weeks) | An influence of training methodology was shown in relation to physical and physiological demands. Thus, both external and internal load must measure to achieve a global vision of athletes’ efforts. | PL  Skills: 351±150  Conditioning: 232±81 | 93.3% |
| [175] | Training | ♂ | Professional | 21 | Rugby union | Optimeye S4 (Catapult Sports) Scapulae | 3D-Accel  100 Hz  Reliable: No  Valid: Yes | PL | One competitive season | The findings show that from the four TL measures, the majority of an individual’s TL information (1st PC: 55 to 70%) during skills training can be explained by either sRPE (PCL: 0.72 to 0.95), TD (PCL: 0.86 to 0.98) or PlayerLoadTM (PCL: 0.71 to 0.98). HSD was the only variable to relate to the *2nd* PC (PCL: 0.72 to 1.00), which captured additional TL information (+19 to 28%). |  | 86.7% |
| [61] | Competition | ♂ | Elite | 33 | American football | SPI HPU (GPSport) Scapulae | 3D-Accel  100 Hz  Reliable: Yes  Valid: Yes | Impacts | 12 official matches (2014 season) | Significant differences were found between playing positions and defensive/offensive skills. The very low impacts were the most registered. Higher values in very low impacts were performed by defensive back and line-backer. Instead, higher values in severe impacts were received by backs and quarterbacks. These differences indicated a specific profile of demands in each playing position and, for this reason, training load should individualize. | Offensive impacts Wide Receiver  5-6 G: 4093 ± 791  6-6.5 G: 1155 ± 401  6.5-7 G: 172 ± 56  7-8 G: 38 ± 14  8-10 G: 11 ± 5  +10 G: 12 ± 5  Running back  5-6 G: 1929 ± 469  6-6.5 G: 582 ± 184  6.5-7 G: 78 ± 31  7-8 G: 21 ± 10  8-10 G: 9 ± 5  +10 G: 16 ± 8  Quarterback  5-6 G: 2060 ± 241  6-6.5 G: 333 ± 109  6.5-7 G: 44 ± 11  7-8 G: 15 ± 5  8-10 G: 9 ± 5  +10 G: 13 ± 5  Tight End  5-6 G: 2615 ± 725  6-6.5 G: 869 ± 255  6.5-7 G: 175 ± 58  7-8 G: 31 ± 14  8-10 G: 9 ± 4  +10 G: 6 ± 2  Offensive Line  5-6 G: 2732 ± 415  6-6.5 G: 851 ± 222  6.5-7 G: 162 ± 103  7-8 G: 36 ± 18  8-10 G: 13 ± 7  +10 G: 12 ± 6 | 80.0% |
| [56] | Competition | ♂ | University | 31 | American football | MinimaxX S5 (Catapult Sports)  Scapulae | 3D-Accel  100 Hz  Reliable: Yes  Valid: No | PL | 15 weeks (training and competition) divided in Pre-season 1, 2 and 3 and In-season (12 weeks) | Notable results included significantly (p ≤ 0.05) greater PLMax values attributed to preseason 1 compared with PL resulting from all in-season practices, and significantly (p ≤ 0.05) higher cumulative PL reported for preseason 1, 2, and 3 compared with every in-season week. Data from this study augment our understanding of the practice demands experienced by NCAA Division I college football players, and provide scope for the improvement of preseason practice design and physical conditioning strategies for coaches seeking to optimize performance. | Accumulative PL  Pre-Season 1: 3757.5  Pre-Season 2: 3563.9  Pre-Season 3: 1937.7  In-Season: 1412-1624 | 86.7% |
| [176] | Competition | ♂ | Elite | 15 | Rugby union | SPI-PRO XII  (GPSport)  Scapulae | 3D-Accel  100 Hz  Reliable: No  Valid: No | Impacts | 45 official matches | The number of high impacts (>10 g) were 48.0±46.9 and 35.6±28.3 times for the forwards and backs, respectively. All characteristics were significantly different between the forwards and backs (p<0.05). | Impacts (>10g)  Forward: 48.0±46.9  Backs: 35.6±28.3 | 60.0% |
| **Note.** ♂: Male; ♀: Female; CMJ: Counter-movement jump; Collisions: Total number of collisions; Collisions/min: collisions per minute; CV: Coefficient of variation; DOMS: Delayed Onset Muscle Soreness; Dynamic Stress Load: Accelerometer load index related to the weight of impacts; DSL/min: Dynamic Stress Load per minute; Force Load: accelerometer-load in 3 axes; Force Load/min: Force load per minute; HR: Heart rate; Impacts: total number of impacts; Impacts/min: Impacts per minute; Locomotor efficiency: Contribution of MD: Match Day; PL(y) in relation to total PL; PL: PlayerLoad^TM^ (sum of 3-axis); PL(x): PL^TM^ x-axis; PL(y): PL^TM^ y-axis; PL(z): PL^TM^ z-axis; PL/min: PL^TM^ (sum of 3-axis) per minute; PL(x)/min: PL^TM^ x-axis per minute; PL(y)/min: PL^TM^ y-axis per minute; PL(z)/min: PL^TM^ z-axis per minute; PL/meters: PL^TM^ (sum of 3-axis) per meter; PL2D: PL^TM^ in 2-axis; PL2D/min: PL^TM^ in 2-axis per minute; PLslow: PL^TM^ where travel speed is <2 m/s; PLslow/min: PL^TM^slow per minute; RD: Total distance covered per minute; sRPE: Session rated perceived exertion; SSG: Small-sided games; TD: Total distance covered; Total Load: Accelerometer load in 3-axis of movement; VO_2_max: Maximal oxygen consumption. | | | | | | | | | | | | |

**References**

32. Colby MJ, Dawson B, Heasman J, Rogalski B, Gabbett TJ. Accelerometer and GPS-derived running loads and injury risk in elite Australian footballers. J Strength Cond Res. 2014;28:2244–2252.

41. Graham SR, Cormack S, Parfitt G, Eston R. Relationships Between Model Predicted and Actual Match Performance in Professional Australian Footballers During an In-Season Training Macrocycle. Int J Sports Physiol Perform. 2018;13:339-346.

44. Boyd LJ, Ball K, Aughey RJ. Quantifying external load in Australian football matches and training using accelerometers. Int J Sports Physiol Perform. 2013;8:44–51.

45. Gabbett T, Jenkins D, Abernethy B. Physical collisions and injury during professional rugby league skills training. J Sci Med Sport. 2010;13:578–583.

46. Gabbett T. Quantifying the Physical Demands of Collision Sports: Does Microsensor Technology Measure What It Claims to Measure? J Strength Cond Res. 2013;27:2319–2322.

52. Ritchie D, Hopkins WG, Buchheit M, Cordy J, Bartlett JD. Quantification of Training and Competition Load across a Season in an Elite Australian Football Club. Int J Sports Physiol Perform. 2016;11:474–479.

53. Johnston RD, Murray NB, Austin DJ, Duthie G. Peak Movement and Technical Demands of Professional Australian Football Competition. J Strength Cond Res. 2019;Epub:Ahead of Print.

56. Wellman AD, Coad SC, Flynn PJ, Siam TK, McLellan CP. Comparison of Preseason and In-Season Practice and Game Loads in National Collegiate Athletic Association Division I Football Players: J Strength Cond Res. 2019;33:1020–1027.

57. Cummins C, McLean B, Halaki M, Orr R. Positional Differences in External On-Field Load During Specific Drill Classifications Over a Professional Rugby League Preseason. Int J Sports Physiol Perform. 2017;12:764–776.

58. Johnston RD, Murray NB, Austin DJ. The influence of pre-season training loads on in-season match activities in professional Australian football players. Sci Med Footb. 2019;3:143–149.

60. Gibson NE, Boyd AJ, Murray AM. Countermovement jump is not affected during final competition preparation periods in elite rugby sevens players. J Strength Cond Res. 2016;30:777–783.

61. Wellman AD, Coad SC, Goulet GC, Coffey VG, McLellan CP. Quantification of Accelerometer Derived Impacts Associated With Competitive Games in NCAA Division I College Football Players. J Strength Cond Res. 2017;31:330-338.

79. Gabbett T. Relationship Between Accelerometer Load, Collisions, and Repeated High-Intensity Effort Activity in Rugby League Players: J Strength Cond Res. 2015;29:3424–3431.

80. Gabbett TJ, Seibold AJ. Relationship between tests of physical qualities, team selection, and physical match performance in semiprofessional rugby league players. J Strength Cond Res. 2013;27:3259–3265.

81. McLellan CP, Lovell DI. Neuromuscular responses to impact and collision during elite rugby league match play. J Strength Cond Res. 2012;26:1431–1440.

82. Suárez-Arrones LJ, Portillo LJ, González-Ravé JM, Muñoz VE, Sanchez F. Match running performance in Spanish elite male rugby union using global positioning system. Isokinet Exerc Sci. 2012;20:77–83.

87. Read DB, Jones B, Phibbs PJ, Roe GAB, Darrall-Jones JD, Weakley JJS, et al. Physical Demands of Representative Match-Play in Adolescent Rugby Union: J Strength Cond Res. 2017;31:1290–1296.

90. Mooney M, Cormack S, O’Brien B, Coutts AJ. Do physical capacity and interchange rest periods influence match exercise-intensity profile in Australian football? Int J Sports Physiol Perform. 2013;8:165–172.

92. Cormack SJ, Mooney MG, Morgan W, McGuigan MR. Influence of neuromuscular fatigue on accelerometer load in elite Australian football players. Int J Sports Physiol Perform. 2013;8:373–378.

93. Gallo T, Cormack S, Gabbett T, Williams M, Lorenzen C. Characteristics impacting on session rating of perceived exertion training load in Australian footballers. J Sports Sci. 2015;33:467–475.

94. Roe G, Halkier M, Beggs C, Till K, Jones B. The Use of Accelerometers to Quantify Collisions and Running Demands of Rugby Union Match-Play. Int J Perform Anal Sport. 2016;16:590–601.

96. Cunniffe B, Proctor W, Baker JS, Davies B. An evaluation of the physiological demands of elite rugby union using global positioning system tracking software. J Strength Cond Res. 2009;23:1195–1203.

110. Johnston RD, Gabbett TJ, Jenkins DG, Hulin BT. Influence of physical qualities on post-match fatigue in rugby league players. J Sci Med Sport. 2015;18:209–213.

111. Rowell AE, Aughey RJ, Clubb J, Cormack SJ. A Standardized Small Sided Game Can Be Used to Monitor Neuromuscular Fatigue in Professional A-League Football Players. Front Physiol. 2018;9:1011.

112. Phibbs PJ, Jones B, Read DB, Roe GAB, Darrall-Jones J, Weakley JJS, et al. The appropriateness of training exposures for match-play preparation in adolescent schoolboy and academy rugby union players. J Sports Sci. 2018;36:704–709.

142. Bayliff GE, Jacobson BH, Moghaddam M, Estrada C. Global Positioning System Monitoring of Selected Physical Demands of NCAA Division I Football Players During Games: J Strength Cond Res. 2019;33:1185–1191.

143. Carey DL, Ong K, Whiteley R, Crossley KM, Crow J, Morris ME. Predictive Modelling of Training Loads and Injury in Australian Football. Int J Comput Sci Sport. 2018;17:49–66.

144. Coad S, Gray B, McLellan C. Seasonal Analysis of Mucosal Immunological Function and Physical Demands in Professional Australian Rules Footballers. Int J Sports Physiol Perform. 2016;11:574–580.

145. Coad S, Gray B, Wehbe G, McLellan C. Physical Demands and Salivary Immunoglobulin a Responses of Elite Australian Rules Football Athletes to Match Play. Int J Sports Physiol Perform. 2015;10:613–617.

146. Cummins C, Welch M, Inkster B, Cupples B, Weaving D, Jones B, et al. Modelling the relationships between volume, intensity and injury-risk in professional rugby league players. J Sci Med Sport. 2019;22:653–660.

147. Davies MJ, Young W, Farrow D, Bahnert A. Comparison of agility demands of small-sided games in elite Australian football. Int J Sports Physiol Perform. 2013;8:139–147.

148. Esmaeili A, Hopkins WG, Stewart AM, Elias GP, Lazarus BH, Aughey RJ. The Individual and Combined Effects of Multiple Factors on the Risk of Soft Tissue Non-contact Injuries in Elite Team Sport Athletes. Front Physiol. 2018;9:1280.

149. Fleay B, Joyce C, Banyard H, Woods CT. Manipulating Field Dimensions During Small-sided Games Impacts the Technical and Physical Profiles of Australian Footballers: J Strength Cond Res. 2018;32:2039–2044.

150. Gallo TF, Cormack SJ, Gabbett TJ, Lorenzen CH. Pre-training perceived wellness impacts training output in Australian football players. J Sports Sci. 2016;34:1445–1451.

151. Gastin PB, McLean O, Spittle M, Breed RVP. Quantification of tackling demands in professional Australian football using integrated wearable athlete tracking technology. J Sci Med Sport. 2013;16:589–593.

152. Garrett J, Graham SR, Eston RG, Burgess DJ, Garrett LJ, Jakeman J, et al. A Novel Method of Assessment for Monitoring Neuromuscular Fatigue Within Australian Rules Football Players. Int J Sports Physiol Perform. 2019;14:598–605.

153. Goodale TL, Gabbett TJ, Tsai M-C, Stellingwerff T, Sheppard J. The Effect of Contextual Factors on Physiological and Activity Profiles in International Women’s Rugby Sevens. Int J Sports Physiol Perform. 2017;12:370–376.

154. Govus AD, Coutts A, Duffield R, Murray A, Fullagar H. Relationship Between Pretraining Subjective Wellness Measures, Player Load, and Rating-of-Perceived-Exertion Training Load in American College Football. Int J Sports Physiol Perform. 2018;13:95–101.

155. Highton J, Mullen T, Norris J, Oxendale C, Twist C. The Unsuitability of Energy Expenditure Derived From Microtechnology for Assessing Internal Load in Collision-Based Activities. Int J Sports Physiol Perform. 2017;12:264–267.

156. Hogarth LW, Burkett BJ, McKean MR. Influence of Yo-Yo IR2 Scores on Internal and External Workloads and Fatigue Responses of Tag Football Players during Tournament Competition. Fisher G, editor. PLOS ONE. 2015;10:e0140547.

157. Johnston RD, Devlin P, Wade JA, Duthie GM. There Is Little Difference in the Peak Movement Demands of Professional and Semi-Professional Rugby League Competition. Front Physiol. 2019;10:1285.

158. Jones M, West D, Crewther B, Cook C, Kilduf L. Quantifying positional and temporal movement patterns in professional rugby union using global positioning system. Eur J Sport Sci. 2015; 15:488-496.

159. Kempton T, Sullivan C, Bilsborough JC, Cordy J, Coutts AJ. Match-to-match variation in physical activity and technical skill measures in professional Australian Football. J Sci Med Sport. 2015;18:109–113.

160. Li RT, Salata MJ, Rambhia S, Sheehan J, Voos JE. Does Overexertion Correlate With Increased Injury? The Relationship Between Player Workload and Soft Tissue Injury in Professional American Football Players Using Wearable Technology. Sports Health Multidiscip Approach. 2020;12:66–73.

161. McLaren SJ, Weston M, Smith A, Cramb R, Portas MD. Variability of physical performance and player match loads in professional rugby union. J Sci Med Sport. 2016;19:493–497.

162. Murray A, Buttfield A, Simpkin A, Sproule J, Turner AP. Variability of within-step acceleration and daily wellness monitoring in Collegiate American Football. J Sci Med Sport. 2019;22:488–493.

163. Owen SM, Venter RE, du Toit S, Kraak WJ. Acceleratory match-play demands of a Super Rugby team over a competitive season. J Sports Sci. 2015;33:2061–2069.

164. Phibbs PJ, Jones B, Roe GA, Read DB, Darrall-Jones J, Weakley JJ, et al. We know they train, but what do they do? Implications for coaches working with adolescent rugby union players. Int J Sports Sci Coach. 2017;12:175–182.

165. Phibbs PJ, Jones B, Roe G, Read DB, Darrall-Jones J, Weakley J, et al. Organized Chaos in Late Specialization Team Sports: Weekly Training Loads of Elite Adolescent Rugby Union Players. J Strength Cond Res. 2018;32:1316–1323.

166. Read DB, Jones B, Phibbs PJ, Roe GAB, Darrall-Jones J, Weakley JJS, et al. The physical characteristics of match-play in English schoolboy and academy rugby union. J Sports Sci. 2018;36:645–650.

167. Roe G, Darrall-Jones J, Till K, Phibbs P, Read D, Weakley J, et al. The effect of physical contact on changes in fatigue markers following rugby union field-based training. Eur J Sport Sci. 2017;17:647–655.

168. Rowell AE, Aughey RJ, Hopkins WG, Stewart AM, Cormack SJ. Identification of Sensitive Measures of Recovery After External Load From Football Match Play. Int J Sports Physiol Perform. 2017;12:969–976.

169. Sullivan C, Bilsborough JC, Cianciosi M, Hocking J, Cordy J, Coutts AJ. Match score affects activity profile and skill performance in professional Australian Football players. J Sci Med Sport. 2014;17:326–331.

170. Tee JC, Coopoo Y, Lambert M. Pacing characteristics of whole and part-game players in professional rugby union. Eur J Sport Sci. 2019;Epub:ahead of print.

171. Twist C, Highton J, Daniels M, Mill N, Close G. Player Responses to Match and Training Demands During an Intensified Fixture Schedule in Professional Rugby League: A Case Study. Int J Sports Physiol Perform. 2017;12:1093–1099.

172. Ward PA, Ramsden S, Coutts AJ, Hulton AT, Drust B. Positional Differences in Running and Nonrunning Activities During Elite American Football Training: J Strength Cond Res. 2018;32:2072–2084.

173. Weaving D, Marshall P, Earle K, Nevill A, Abt G. Combining Internal- and External-Training-Load Measures in Professional Rugby League. Int J Sports Physiol Perform. 2014;9:905–912.

174. Weaving D, Jones B, Marshall P, Till K, Abt G. Multiple Measures are Needed to Quantify Training Loads in Professional Rugby League. Int J Sports Med. 2017;38:735–740.

175. Weaving D, Dalton NE, Black C, Darrall-Jones J, Phibbs PJ, Gray M, et al. The Same Story or a Unique Novel? Within-Participant Principal-Component Analysis of Measures of Training Load in Professional Rugby Union Skills Training. Int J Sports Physiol Perform. 2018;13:1175–1181.

176. Yamamoto H, Takemura M, Iguchi J, Tachibana M, Tsujita J, Hojo T. In-match physical demands on elite Japanese rugby union players using a global positioning system. BMJ Open Sport Exerc Med. 2020;6:e000659.
