# Supplementary material for: Accelerometry as a method for external workload monitoring in invasion team sports. A systematic review
Source: PLoS One. 2020 Aug 25;15(8):e0236643. doi: 10.1371/journal.pone.0236643 (PMC7447012; doi:10.1371/journal.pone.0236643)
Supplement: S4 Table — (DOCX) [file pone.0236643.s004.docx]

| **S4 Table.** *Quality criteria used to analyze the quantitative publications* (extracted from Law et al. [37]). | | | |
| --- | --- | --- | --- |
| *Q1* | Was the study purpose stated clearly? | 1=Yes | 0=No |
| *Q2* | Was relevant background literature reviewed? | 1=Yes | 0=No |
| *Q3* | Was the design appropriate for the research question? | 1=Yes | 0=No |
| *Q4* | Was the sample described in detail? | 1=Yes | 0=No |
| *Q5* | Was sample size justified? | 1=Yes | 0=No |
| *Q6* | Was informed consent obtained? (if not described, assume No) | 1=Yes | 0=No If not applicable, assume N/A |
| *Q7* | Were the outcome measures reliable? (if not described, assume No) | 1=Yes | 0=No |
| *Q8* | Were the outcome measures valid? (if not described, assume No) | 1=Yes | 0=No |
| *Q9* | Was method described in detail? | 1=Yes | 0=No |
| *Q10* | Were results reported in terms of statistical significance? | 1=Yes | 0=No |
| *Q11* | Were the analysis methods appropriate? | 1=Yes | 0=No |
| *Q12* | Was importance for the practice reported? | 1=Yes | 0=No |
| *Q13* | Were any drop-outs reported? | 1=Yes | 0=No If not applicable, assume N/A |
| *Q14* | Were conclusions appropriate given the study methods? | 1=Yes | 0=No |
| *Q15* | Are there any implications for practice given the results of the study? | 1=Yes | 0=No |
| Q16 | Were limitations of the study acknowledged and described by the authors? | 1=Yes | 0=No |
