# Supplementary material for: Accelerometry as a method for external workload monitoring in invasion team sports. A systematic review
Source: PLoS One. 2020 Aug 25;15(8):e0236643. doi: 10.1371/journal.pone.0236643 (PMC7447012; doi:10.1371/journal.pone.0236643)
Supplement: Attachment — Submitted filename: Response_Reviewers.pdf [file pone.0236643.s007.pdf]

Dear Editor,

We have carefully considered all reviewers' considerations of the paper (PONE-D-19-33961). Please find enclosed our detailed answers to reviewers' queries. The authors declare that the manuscript is original and has not been considered for publication elsewhere. Additionally, the authors had approved the paper for release and agree with its content.

Journal Requirements

- **C1.** *Please ensure that your manuscript meets PLOS ONE's style requirements, including those for file naming. The PLOS ONE style templates can be found at http://www.plosone.org/attachments/PLOSOne_formatting_sample_main_body.pdf and http://www.plosone.org/attachments/PLOSOne_formatting_sample_title_authors_affiliations.pdf*

  Thanks for your appreciations. The authors are ensured that our manuscript meets PLoS One's style requirements, including those for file naming.

- **C2.** *PLOS ONE does not permit references to unpublished data; therefore, we request that you either include the referenced data or remove the instances of "data not shown," "unpublished results," or similar.*

  Yes, we do agree with your comment. The references to unpublished data were removed and cited in the new version of the manuscript.

- **C3.** *In your Data Availability statement, you have not specified where the minimal data set underlying the results described in your manuscript can be found. PLOS defines a study's minimal data set as the underlying data used to reach the conclusions drawn in the manuscript and any additional data required to replicate the reported study findings in their entirety. All PLOS journals require that the minimal data set be made fully available. For more information about our data policy, please see http://journals.plos.org/plosone/s/data-availability.*

  Thanks for your suggestion. The data set to reach the conclusions drawn in the manuscript and to replicate the reported study findings was attached in the new submission after peer-review process.

- **C4.** *Please include your tables as part of your main manuscript and remove the individual files. Please note that supplementary tables (should remain/ be uploaded) as separate "supporting information" files*

  We appreciate your suggestion. The table was included as part of the manuscript and removed the individual file.

Reviewer 1

General comments

- **R1_GC1.** *This systematic review provides insights regarding the use of accelerometers to quantify external workloads in team sports. The literature review is well-written and*

correctly formatted for submission to PLOS ONE; it is well organized providing clear and comprehensive information on the topic. A thorough analysis of current literature is provided identifying and discussing gaps on this topic; however, the introduction section should be improved to further underline the need to develop this systematic review. The current version of the manuscript clearly presents the literature background, without really pointing out why this review is needed. Furthermore, authors should develop the discussion section to point out the relevant information retrieved for practitioners. Please make sure to really edit the manuscript according to these overall suggestions. Furthermore, a better use of abbreviations is required throughout the manuscript. Specific comments are listed below.

The authors really appreciate all the suggestions made to improve the quality of the manuscript. All of them have been carried out following your considerations.

Specific comments

First page

- **R1_SC1.** Line 11 – “associate professor” should not be included here.

  Thanks for your comment. “Associate professor” was deleted.

Abstract

- **R1_SC2.** L41 – replace “colon” with “comma”.

  Thanks for your suggestion. Colon was used throughout the sentence to separate the most frequent findings in the systematic review.

Introduction

- **R1_SC3.** L50 – Be consistent with the use of “load” or “workload” throughout the manuscript. Edit accordingly within the entire manuscript.

  We really appreciate your suggestion. The term “workload” was used throughout the manuscript.

- **R1_SC4.** L60 – Please replace “rated” with “a rating of”.

  Thanks for your suggestion. “Rated” was changed by “a rating of”.

- **R1_SC5.** L61 – Remove “on the other hand”. This is too colloquial.

  Yes, we do agree with your comment. The term “on the other hand” was changed by “In contrast”.

- **R1_SC6.** L62 – Include “various variables like” after “through”.

  Thanks for your proposal. “Various variables like” was included after “through”.

- **R1_SC7.** L63-64 – Please try to link the first and second paragraphs of the introduction section. It appears that you completely.

  Thanks for your suggestion. A sentence was added to link the first and second paragraphs.

- **R1_SC8.** L70 – Specify what are these instruments considered as "gold standard" and "criterion measures".

  We really appreciate your suggestion. A sentence was added to explain which are these instruments and applications for workload monitoring.

- **R1_SC9.** L82 – Please specify that you are referring to the "internal" load.

  Thanks for your comment. The word "internal" was added before "workload".

- **R1_SC10.** L84-85 – This sentence is not clear. Please edit for clarity.

  Yes, we do agree with your comment. The sentence was rephrased to improve its clarity.

- **R1_SC11.** L89 – Include the following reference here. A Review of Player Monitoring Approaches in Basketball: Current Trends and Future Directions. Fox JL, Scanlan AT, Stanton R. J Strength Cond Res. 2017 Jul;31(7):2021-2029. doi: 10.1519/JSC.0000000000001964.

  Thanks for your suggestion. The citation was included.

- **R1_SC12.** L93 – "rated perceived exertion" should be "RPE". Be consistent when using abbreviations within the manuscript and check the other abbreviations used. Make sure to explain it the first time you use it and then use the abbreviation.

  We really appreciate your suggestion and we agree with it. RPE was utilized throughout the manuscript, except the first appearance in the introduction section.

- **R1_SC13.** L94-96 – A reference is required here.

  Thanks for your suggestion. The citation was included.

- **R1_SC14.** L96-99 – This is a very important point to be discussed here. Please consider also to include something about the comparison between accelerometers and time-motion analysis. With this regard, accelerometers do not provide information about static actions too when an effort is performed without an acceleration (e.g. screenings or a prolonged stance position in basketball) but their reliability is greater compared to other systems. Furthermore, accelerometers provide real-time data. This paragraph should be improved to better develop the importance of using accelerometers compared to other systems and to further underline the importance of carrying out this review. For example, it has been stated that "microtechnologies (e.g., wearable microsensors and accelerometers) in basketball may represent a valid and practical alternative to TMA" (Ferioli, 2020) or "accelerometers offer

distinct advantages compared with TMA, with accelerometer data being relatively simple to analyze using either proprietary or user-defined algorithms that quantify movement" (Fox, 2017).

- o Match Activities in Basketball Games: Comparison Between Different Competitive Levels. Ferioli D, Schelling X, Bosio A, La Torre A, Rucco D, Rampinini E. J Strength Cond Res. 2020 Jan;34(1):172-182. doi: 10.1519/JSC.0000000000003039.
- o A Review of Player Monitoring Approaches in Basketball: Current Trends and Future Directions. Fox JL, Scanlan AT, Stanton R. J Strength Cond Res. 2017 Jul;31(7):2021-2029. doi: 10.1519/JSC.0000000000001964.

We really appreciate your suggestion and have been used to improve the final version of the manuscript. The last paragraph of the introduction section has been improved following your recommendations and citations provided.

- **R1_SC15.** L115 - Should "retrieved" be a "peer-reviewed" manuscript?

Thanks for your proposal. "Retrieved" was changed by "peer-reviewed".

- **R1_SC16.** L128 - Why did the authors include only two electronic databases? The inclusion of other databases (like Scopus, MEDLINE, ERIC, Google Scholar, SCIndex, ScienceDirect..) would have been more appropriate.
- **R1_SC17.** L130 – In line with my previous comment, including more keywords (e.g. microtechnologies, workloads, physical demands) would have been more appropriate.

Thanks for your proposals. Your considerations were carried out. For this reason, authors have decided to review more databases and include the keywords according to your suggestions. All sections have been improved according to the new studies included.

This manuscript is a systematic review [32] about peer-reviewed manuscripts (scientific papers) related to workload monitoring via accelerometry in sports. The Web of Science (Web of Science Core Collection, MEDLINE, Current Contents Connect, Derwent Innovations Index, KCI-Korean Journal Database, Russian Science Citation Index and Scielo Citation Index), PubMed electronics and Scopus electronic databases were searched on 1st May 2020 for relevant articles published between 1st January 2010 and 30th April 2020 using the keywords "accelerometer" or "accelerometry" or "microtechnology" or "inertial devices", and "load" or "workload", and "sport".

- **R1_SC18.** L140 – Replace (iii) with "(3)"
- **R1_SC19.** L141 – Replace (3) with "(4)"

Thanks for your comments. The paragraph was improved following the recommendations R2_SC18, R2_SC19, and R2_SC22.

- **R1_SC20.** Figure 2b – "Int J Sport Physiol" should be "Int J Sports Physiol Perform"; "J Sport Sci" should be "J Sports Sci". Please check all the journal abbreviations and edit them if needed.

Thanks for your appreciation. Figure 2b was improved accordingly.

- **R1_SC21.** L261-263 – The abbreviations included here have not been explained before. Consider including the variables without abbreviation and/or include something like "see Table 1 for definitions".

  Thanks for your suggestion. "see Table 1 for definitions" was added at the end of the paragraph.

- **R1_SC22.** L273 – "PlayerloadTM" should be PLTM. (see line 244). The authors should check the use of correct abbreviations (when required) throughout the manuscript.

  Thanks for your comment. PlayerLoad^{TM} was changed by PL^{TM} from its first appearance.

- **R1_SC23.** L307-312 – I would suggest including a message for practitioners regarding the use of accelerometers when monitoring workloads during training or competitions. The current version of this paragraph does not provide any relevant information, while this systematic review should provide insights on this topic. For example, is it important to monitor external workloads during both competitions and training? Would the monitoring of competition workloads assist in planning training workloads? The authors did a big effort in investigating workload sustained during both competitions and training, as such, they should provide some relevant information here to underline the utility of this systematic review.

  Thanks for your proposal. The last paragraph of the competition vs training section of the discussion section was modified according to your suggestion, including the advice for practitioners regarding the use of accelerometers when monitoring workloads during training or competition.

- **R1_SC24.** L320 – definitions of GPS and GNSS are required here.
- **R1_SC25.** L323 – definitions of LPM and UWB are required here.

  Thanks for your suggestions. The paragraph was rewritten and the definitions of tracking systems were incorporated.

- **R1_SC26.** L337 – definition of HUB is required here.

  Yes, we do agree with your comment. The definition of the HUB was incorporated.

- **R1_SC27.** L331-342. This paragraph is well discussed, providing relevant information and practical applications. Well done.

  We really appreciate your comments. The authors want to thank your suggestions throughout the whole manuscript.

- **R1_SC28.** L340 and 344 – Neuromuscular load – Here the authors are including the term "neuromuscular load" that was not included in the introduction or elsewhere before. Can this term be replaced with the external workload as done in the introduction? If not, the introduction should be edit accordingly.

Thanks for your comment. The term “neuromuscular load” was replaced by “accelerometry-based workload” or “external workload” as you proposed.

- **R1_SC29.** L350-351 – Are the authors referring to soccer competitions? This is not clear.

  Yes, we do agree with your suggestion. The sentence was rewritten for better comprehension.

- **R1_SC30.** L359 – Again, “PlayerloadTM” should be PLTM. (see line 244).

  Following your previous recommendation R1_SC22, the index was abbreviated.

- **R1_SC31.** L381-382 – “making possible objective players’ weaknesses detection” is not clear enough. Please edit for clarity.

  Thanks for your suggestion. The sentence was edited for clarity.

- **R1_SC32.** L390-392 – This is a systematic review, as such unpublished data should not be included. Remove this sentence. It might be more appropriate to include something regarding the development of a strategy to correctly use and interpret data retrieved with different systems (and algorithms).

  Thanks for your suggestion. Current published research related to the study topic was added.

- **R1_SC33.** L416 – Include the following reference here: The relationships between internal and external training load models during basketball training. Scanlan AT, Wen N, Tucker PS, Dalbo VJ. J Strength Cond Res. 2014 Sep;28(9):2397-405. doi: 10.1519/JSC.0000000000000458.

  Thanks for your recommendation. The citation was added as you suggested.

- **R1_SC34.** L417 – RPE was already abbreviated.

  Following your previous recommendation R1_SC12, the index was abbreviated.

- **R1_SC35.** L413-423 – The authors should consider that despite the existing associations, accelerometers are used to quantify the external workloads, while the cited tools are used to measure the internal workloads. This should be underlined, as the reader could erroneously interpret that the systems are interchangeable. This is not the case, as workload quantification should include both internal and external measures. It might be of interest to include here and discuss the methodologies used to quantify external workloads (TMA, tracking systems..) highlighting the positive and negative aspects of using accelerometers than other systems. This paragraph should be edited accordingly.

  We really appreciate your suggestion. The paragraph was rewritten following your considerations.

<u>**Reviewer 2**</u>

General comments

- **R2_GC1.** *The manuscript seems to fulfill the journal publication criteria and addresses a topic perceived relevant by the scientific community for training monitoring and regulation. To a large extent, the manuscript documents descriptive data of the selected studies. The interpretation and conclusions for practical relevance can be improved. Otherwise, the value of this review is questionable. A good example of a clearly outlined relevance of this manuscript's contribution to the state of knowledge and community is device location. Here, the manuscript clearly can recommend the most common and best practice. In other regards, the value added by this review (except for the documentation of descriptive numbers) seems often questionable. In general, the value of the reported numbers can be improved by deepening the interpretation and conclusions drawn from these numbers.*

  We really appreciate your comments. The manuscript has been improved according to your proposals.

- **R2_GC2.** *A second general concern is a way how the relevance of this review and objectives is displayed in the introduction. It is recommended to outline the current deficits and needs that justify this review rather than building strongly on an increased interest (see L100), which is, by the way, one of the results and not a given fact at the stage of the introduction (at least it is not supported by the literature). This makes it a weak justification.*

  Yes, we do agree with your suggestions. The last paragraph of the introduction was modified in this sense.

- **R2_GC3.** *Other general concerns are addressed in specific comments relating to concrete examples but should be considered throughout the manuscript. The whole manuscript requires to be checked for weaknesses comparable with the examples pointed out in the specific comments.*

  Thanks for your proposals. All specific comments have been considered to improve the final version of the manuscript.

- **R2_GC4.** *The manuscript can be suitable for further consideration after a major revision. Perhaps, an expert in scientific English writing should be consulted.*

  The authors considered your suggestions and the manuscript has been proofread by an English service, specialist in the sports science area.

Specific comments

Abstract

- **R2_SC1.** *L30-31: „for using it if they are coherently organized" is no valuable information. More valuable would be to add the benefit of applying the method. For example: "A rapidly increasing number of studies supports the practical implementation of accelerometry monitoring to regulate and optimize training schemes." (If such statement is in line with the literature and the authors' intentions).*

  Thanks for your comment. The sentence was modified as you suggested.

- **R2_SC2.** *L33: "and identify and organize them": First, correct is "TO identify and organize them". Second, these are weak objectives. Consider something like "to reflect the current state of knowledge and to conclude recommendations for application and scientific investigations".*

  Yes, we do agree with your comment. The objectives were modified to reflect the importance of the study following the reviewer's suggestions.

- **R2_SC3.** *L38-39: The subsequent list is not a list of "fulfilled aspects". Reword (e.g. "main findings include"). The list is also unclear; it is not self-explaining that the first item is the addressed issue (e.g. body location for device), followed by the specification/most frequent finding, etc. (e.g. scapulae). Also, the punctuation marks are inconsistent (i.e. "," vs ":").*

  Thank you very much for your positive feedback. The start of the sentence was reworded to self-explaining the addressed issue and the most frequent finding. The punctuation marks were modified to be consistent (choose ":" throughout the abstract).

- **R2_SC4.** *L42-44: Please aim for condensed language, straight to the point: E.g. "A great number of variables and devices from various companies makes comparability between findings difficult; unification is required." Also, be careful with terms like "evident".*

  We really appreciate your suggestions and we agree with them. The sentence mentioned and the next sentence were condensed as you suggested to straight to the point.

Introduction

- **R2_SC5.** *L52-54: First, no "," before "that". Second, the information "that is analyzed by sports scientists and fitness coaches" is not very relevant and can be removed. Third, "to explain" can be replaced by "to explain". Fourth, "explaining training effects": Really? Maybe more appropriate would be "regulate training volumes" or something comparable. Fifth, "injury risk or that of overtraining": Reword as "risk of injuries and overtraining". Please take this first sentence as an example of how to improve the writing throughout the whole manuscript.*

  Thanks for your suggestions. All of them have been considered to improve the first sentence of the manuscript.

- **R2_SC6.** L55: "respond" is correct.

  Yes, I agree with your comment.

- **R2_SC7.** L64: Be more precise. It is not an area. Consider "At high levels"

  Thanks for your comment. The text was modified according to your proposal.

- **R2_SC8.** L65: Shorten: "to obtain" instead of "to obtain"

  Thanks for your comment. The text was modified according to your proposal.

- **R2_SC9.** L66-67: The reviewer thinks that it is not the performance that is difficult to be measured but developments, training activity, input-outcome, etc. Consider rewriting this statement because, as it is, this is not the reason for the subsequent statement (i.e. different methods to measure workloads indirectly).

  We really appreciate your suggestion. The sentence has been rewritten in the new version of the manuscript.

- **R2_SC10.** L77: Do not use "among others". Consider "include A, B, C". Then, there is no need for "among others", "and more", "etc.".

  Yes, we do agree with your comment. The inclusion of A, B, C was realized and the term "among others" was deleted.

- **R2_SC11.** L79: no "," before "such".

  Thanks for your suggestion. The comma was deleted.

- **R2_SC12.** L100: the "increasing interest" is not a given fact at this stage and is not supported by the literature. It is a part of the result of this study. So, please do not refer to it as a reason for this study. Instead, you may reason by benefits of accelerometry over other methods as you indicated in the comparison with video tracking methods.
- **R2_SC13.** L101 ff: Please shorten the purpose and focus on things that are relevant to clarify what the major purpose is. Do not redundantly use 2 sentences introducing different purposes. The purpose and objective should match the need analysis for this study (i.e. be in line with its relevance). For example, the purpose is to summarize/reflect the current state, outline best practices, and conclude recommendations.

  We really appreciate your suggestions. All of them have been considered to improve the final version of the manuscript.

Methods

- **R2_SC14.** L113-116: Another example for condensing the manuscript: Please consider to merge both sentences into "This manuscript is a systematic review that analyses [30] scientific articles related to workload monitoring via accelerometry in sports." Note that you downgrade your work by stressing it is an exclusively theoretical work.
- **R2_SC15.** L116-118: Please remove completely. Repeating systematic methodology is self-explaining in a systematic review and having no complex statistics is again downgrading your work. There is no point in doing such a statement.
- **R2_SC16.** L119-125: Such questions do not belong in this chapter; they should be clarified in the introduction. The reviewer thinks they are clear. Second, they are not required for the justification of the inclusion criteria. Therefore, the reviewer suggests removing this part. This allows merging this sub-chapter with the subsequent one: One chapter "Design and Search Strategy". You may start with the sentence suggested in the previous comment and then go on with L128 introducing the database search.

  We thank so much for these suggestions. Firstly, respect to R2_SC14 the text was modified accordingly to your proposal. Second, the text was removed completely according to

R2_SC15. Finally, respect to R2_SC16, the subchapter Study design, and Search strategy were merged in one chapter with the title that you proposed.

- **R2_SC17.** L132: Replace “or” by “and”

  Thanks for your comment. “Or” was replaced by “and” as you suggested.

- **R2_SC18.** L139: Consider “across ages” or replace “different” with “various”. Otherwise, “different” suggests that it was a criterion to have at least 2 levels in each study and these levels must differ. Maybe the best would be to simply remove it. As the reviewer understood it, the level was not a criterion. Studies were included “irrespective of level” (that could be another wording you may use).

  Yes, we do agree with your comment. The term “irrespective of level” was employed following your considerations.

- **R2_SC19.** L140: Same goes for sexes (see the previous comment). Both sexes separately and the combined analyses of both were included in the review. So, sex is no inclusion/exclusion criterion. Also, what is this different format of the number (i.e. “iii”)?

  Thanks for your consideration. The criteria of R2_SC18 and the criteria of R2_SC19 were merged as one inclusion criteria: “(2) participants were healthy players irrespective of level (amateur, well-trained, professional, elite, junior, senior) and genres (male and female)”. The number “iii” was deleted.

- **R2_SC20.** L150: Please adjust by saying “competitive sports context” because it would be wrong to say health activities and practices implemented in the elderly cannot be a sport! Please remove “older people” in brackets.

  Thanks for your proposal. The sentence was changed following your proposal: “(4) the study context was outside competitive sports”. The terms in brackets were deleted.

- **R2_SC21.** L152: Remove the sixth point. It is redundant after defining invasive team sports as an inclusion criterion previously.

  Yes, we do agree with your comment. The sixth point was deleted because it is redundant.

- **R2_SC22.** L158: Please change “competition (playing level and sport)” to “sport and competition level”. Moreover, check throughout the rest of the manuscript to use “competition level” instead of players’/playing/sports level.

  Thanks for your suggestion. All changes were made as you proposed.

- **R2_SC23.** L168-170: Please shorten to “by five university full professors with a PhD in sports science and a large number of publications in the field of xy [specify; 1-2 words].”

  We really appreciate your suggestion. The sentence was shorten following your proposals.

- **R2_SC24.** L176: Do you mean “rated” instead of “scored”?

Thanks for your comment. “Scored” was changed by “rated”.

Results

- **R2_SC25.** L192: You may shorten as follows: “From the remaining 296 articles, 83 did not fulfill the inclusion criteria and were removed after revision of the abstract and another 141 after full-text assessment.”

  Thanks for your consideration. The sentences were shortened as you proposed.

- **R2_SC26.** L201: Consider to shorten: “The main reasons for exclusion were non-competitive sports contexts (n=41) and reliability and validity analyses of raw data and load indexes (n=37)”.

  Thanks for your proposal. The sentence was rewritten as you proposed.

- **R2_SC27.** L203-205: If these two reasons’ numbers are mentionable, please add the numbers (n=?). Otherwise, there is no need to mention these two reasons at all.

  Yes, we do agree with your comment. The n values were added in these two reasons.

- **R2_SC28.** L210-217: The usage of Kappa, thresholds for quality levels, and 95% CI should be introduced in the methods section.

  We really appreciate your suggestion. The usage of Kapa, the threshold for quality levels, and 95% CI were introduced in the methods section, sub-section Quality of the studies.

- **R2_SC29.** L232: Remove “Concerning the sports context”. Just start with “Most studies […]”.

  Thanks for your comment. “Concerning the sports context” was deleted as you suggested.

- **R2_SC30.** L237: Change to “males” and “females” instead of “men” and “women” as you mention that some analyzed juniors.

  Thanks for your suggestion. Men and women were changed by males and females.

- **R2_SC31.** L244: Most FREQUENTLY used.

  Thanks for your proposal. The word “frequently” was added.

- **R2_SC32.** L259-260: Again, remove “with respect…”. Change to “x, y, and z were the most frequently investigated invasion team sports.”

  Yes, we do agree with your comment. The sentence was modified according to your proposal.

- **R2_SC33.** L260: What do you mean by “in a specific manner”?

Thanks for your comment. The term “in a specific manner” was changed by “Also”.

Discussion

- **R2_SC34.** L267: As mentioned in general comments: to “select” and describe is a weak purpose. Consider “summarise” and something like “identify common practices and conclude recommendations” based on the review. For explanation: A pure selection and reflection of descriptive numbers is not very helpful for the community except for other manuscripts to back up statements like “commonly/most frequently used”. A more valuable contribution is to draw conclusions and to outline recommendations based on these numbers.

  Thank you very much for the proposal. The purposes of the study were changed according to abstract and introduction following the suggestions R2_SC1, R2_SC12 y R2_SC13.

- **R2_SC35.** L269: Replace “Thus” with “Therefore”. Please be aware that they are not interchangeable as thus strongly relates to the way how something is done.

  Yes, we do agree with your comment. “Thus” was replaced by “Therefore”.

- **R2_SC36.** L271: Be precise and correct in language: The evolution is not rapid. The increase may be. Here, the reviewer suggests “show a rapidly increasing number of publications”

  We really appreciate your suggestions and we agree with them. The sentence was modified according to your proposal.

- **R2_SC37.** L276: “Most studies” instead of “Most of the studies”

  Thanks for your suggestion. “Most of the studies” were changed by “Most studies”.

- **R2_SC38.** L277: “limited” instead of “reduced”

  Thanks for your suggestion. “reduced” was changed by “limited”.

- **R2_SC39.** L279: “occurrence” instead of “appearance”

  Thanks for your suggestion. “appearance” was changed by “occurrence”.

- **R2_SC40.** L283: Add: a total NUMBER of 16 publications

  This suggestion was nice to you. The word “number” was added between total and of.

- **R2_SC41.** L284-286: Please clarify here if these studies provided training and competition hours so that they allowed for normalization or not. If not, state it here.

  Thank you very much for your positive feedback. The paragraph was improved accordingly.

- **R2_SC42.** L286: Rephrase: “Four studies that compared both sport contexts found higher workload in training than in competition; three articles reported the contrary.”

Thanks for your proposal. The sentence was rephrased.

- **R2_SC43.** L290: Please clarify “in relation to their purpose”

  This suggestion was nice of you. This idea was clarified in the text, and the word “purpose” was changed by “integrated contents”.

- **R2_SC44.** L292: “female” instead of “women”

  Thanks for your suggestion. “Women” was changed by “female”.

- **R2_SC45.** L291+294+302+305: Probably you mean “in comparison with” instead of “with respect to”. Please be aware that these are not interchangeable.

  We really appreciate your suggestion. “With respect to” was changed by “in comparison with” in these two paragraphs.

- **R2_SC46.** L295-298: Please rephrase and condense the findings from Ritchie et al. Writing “they compared a with b, finding higher values in a and lower values in b” is not recommended.

  Thanks for your suggestion, which has used to improve the final version of the manuscript.

- **R2_SC47.** L310: Consider “suitable” instead of “necessary”.

  Thanks for your proposal. “Necessary” was replaced by “suitable”.

- **R2_SC48.** L323: If the reviewer is not mistaken, LPM stands for Local Position Measurement (you did not introduce this and other abbreviations) and is not defined by the system that is applied to identify the location. Please check and correct it in case.

  Thanks for your suggestion, which has used to improve the final version of the manuscript. The sentence was rephrased according to your proposals.

- **R2_SC49.** L334: The reviewer suggests being careful with “must” in this context. Consider “should” or “is recommended”

  Thanks for your suggestion. “Must” was changed by “should”.

- **R2_SC50.** L344: Is it really NEUROMUSCULAR load?

  Yes, we do agree with your comment. “Neuromuscular” was changed by “accelerometry-based”.

- **R2_SC51.** L351: Sentence is unclear. Add units to impact numbers. What means “greater impacts ARE FROM x±y”? Rephrase. Reword “under-19”/”under-15”.

  Thank you very much for the proposal. The sentence was rephrased for better comprehension. The sports categories (under-19, and under-15) were deleted.

- **R2_SC52.** L353-355: Change to “due to the lower intensity of physical contacts in soccer compared with American football”

  Thanks for your comment. The sentence was changed as you proposed.

- **R2_SC53.** L356: “Therefore” instead of “Thus”.

  Thanks for your suggestion. The word was changed as you proposed.

- **R2_SC54.** L359: most FREQUENTLY used. Also, replace “that” by “, which” (with comma)

  Thanks for your suggestion. The word was changed as you proposed.

- **R2_SC55.** L360-367: Rephrase these two sentences. For example: “For comparison between sports disciplines, the variable PLTM is normalized by total session time [min]. Studies that included time normalized PLTM reported different workloads across sports (soccer: x, netball: y…)”

  We really appreciate your suggestion. The sentence was rewritten according to your proposal.

- **R2_SC56.** L372: Reword “which of them most contributes”.
- **R2_SC57.** L375-376: Reword “plane where the movement is produced”.

  Thanks for your proposals. The sentence was rephrased for a better comprehension.

- **R2_SC58.** L378-382: Shorten and clarify the sentence and statements made.

  We really appreciate your suggestion. The sentence was shortened and clarified according to your proposal.

- **R2_SC59.** L386: Shorten to “These indexes”

  Thanks for your comment. The start of the sentence was shorted as you proposed.

- **R2_SC60.** L387: Change “but with the implementation of” to “applying”.

  Thanks for your comment. “but with the implementation of” was changed by “applying”.

- **R2_SC61.** L388: Simplify (e.g. “This makes the comparability of data from different devices difficult. The result is a […]”.

  Yes, we do agree with your comment. The sentence was simplified.

- **R2_SC62.** L440-441: The review did not assess the state of validity and reliability of workload monitoring and cannot draw conclusions if the existent research sufficiently covers this concern, right? You may elaborate this in an appropriate section to allow such a

conclusion. The reviewer recommends such addition rather than removing this conclusion to strengthen the value of the current manuscript.

Yes, we do agree with your comment. A new section in the discussion (Accelerometer technical features) was elaborated to allow the conclusion reported.

- **R2_SC63.** L447: “LPS” seems to be a typo. Probably, “LPM” was meant.

  Thanks for your comment. “LPS” was changed by “LPM”.
