# Supplementary material for: Accelerometry as a method for external workload monitoring in invasion team sports. A systematic review
Source: PLoS One. 2020 Aug 25;15(8):e0236643. doi: 10.1371/journal.pone.0236643 (PMC7447012; doi:10.1371/journal.pone.0236643)
Supplement: Attachment — Submitted filename: Response_Reviewers.pdf [file pone.0236643.s008.pdf]

Dear Editor,

We have carefully considered all reviewers' considerations of the paper (PONE-D-19-33961_R1). Please find enclosed our detailed answers to reviewers' queries. The authors declare that the manuscript is original and has not been considered for publication elsewhere. Additionally, the authors had approved the paper for release and agree with its content.

Reviewer 1

General comments

- **R1_GC1.** *This The authors did a good job editing the manuscript in accordance with my previous comments and implementing the number of scientific databases revised for this systematic review The rationale of the study within the introduction section, the results section and some paragraphs of the discussion section have been better developed. In my opinion, some amendments are still required to improve the quality of the manuscript. In addition, I would suggest a further proofread of the entire manuscript as it still presents some grammatical errors and, in some cases (especially in the discussion section), poorly fluid and clear sentences. Continuous line numbering has not been used, please remember to do this step in your future submissions as stated in the authors' guidelines.*

  The authors really appreciate all your kindly comment related to the previous peer-review step of our manuscript. Besides, we apologize for not including continuous line numbering in the previous revision. For the next revision, all comments have been considered and continuous line numbering was added.

Specific comments

Abstract

- **R1_SC1.** *Page 2, Line 5-7. Please include "1)" and "2)" before each aim*

  Thanks for your comment. 1) and 2) were included before each aim.

- **R1_SC2.** *P2, L18-19. "due to it is" does not sound very fluent. Please edit.*

  Thanks for your suggestion. The sentence was edited to improve its clarity.

Introduction

- **R1_SC3.** *P2 L25, L29-30 "Load" should be "workload". I still noticed the use of "load" in some sentences. Please replace it with "workload" where needed.*

  Thanks for your suggestion. Authors apologize you for the mistake committed in the previous revision. "Load" was replaced by "workload" through the manuscript.

- **R1_SC4.** *P2 L26. "decreasing" should be "to decrease".*

  Thanks for your suggestion. "Decreasing" was changed by "to decrease".

- **R1_SC5.** *P3 L5-7. This sentence should be edited stating that current literature suggests to adopt strategies to monitor and quantify both internal and external workload sustained during training and competitions.*

  Yes, we do agree with your comment. The sentence was modified according to your proposal.

- **R1_SC6.** *P3 L16. I do not agree with authors stating that current methodologies are "without restrictions". This should be removed as this is not true. For example, in some professional basketball leagues the use of microsensors during competitions is not allowed (e.g. basketball).*

  Thanks for your proposal. The term "without restrictions" was removed.

- **R1_SC7.** *P4 L10-15. This sentence is very hard to read and should be edited and/or splitted.*

  Thanks for your suggestion. The sentence was splitted as you recommended.

- **R1_SC8.** *P4 L14-18. I do not fully agree with this statement as TMA in some cases can be more adequate for workload (and not "load" here - edit) quantification. Authors should consider differentiating manual than automatic and semi-automatic systems of TMA. Manual TMA can be used to identify static movements performed with high-intensity efforts (e.g. body contacts) better than accelerometers, while I agree that automatic video-based analysis can underestimate workload. Edit this section accordingly.*

  We really appreciate your suggestion. The sentence was modified according to your proposal.

Results

- **R1_SC9.** *P7 L3. Should "1373" be "1371"? Please check articles numbers in this section.*

  Thanks for your appreciation. The number "1373" was changed by "1371".

Discussion

- **R1_SC10.** *P12 L2-5. Please do not repeat the aim of the study here. Include a general statement to resume what has been included in this systematic review.*

  We really appreciate your suggestion. The sentence was rephrased to include a general statement to resume what has been included in this systematic review.

- **R1_SC11.** *P12 L26. "To possibility" should be "to permit". Please edit.*

  Thanks for your suggestion. "To possibility" was changed by "to allow".

- **R1_SC12.** *P12 L29 to P13 L1-2. The contrasting results may be also a consequence of different weekly schedules adopted. Competition formats (e.g. 1 game per week vs 2-3 games*

per week) considerably affect the workload outcomes, thus I suggest including this reasoning to justify the differences between these studies.

We really appreciate your suggestion. The mention to competition formats as contextual variable that affects workload outcomes was included.

- **R1_SC13.** *P14 L8-9. The abbreviation "GNSS" and "LPM" are required here. Please check the entire manuscript for the use of correct abbreviations.*

  Thanks for your suggestion. The abbreviations GNSS and LPM were used throughout the manuscript.

- **R1_SC14.** *P15 L10. Please edit with something like: the impacts registered >5G range from .. to ..* ; and *"in soccer" is repeated twice.*

  Yes, we do agree with your comment. The sentence was modified accordingly.

- **R1_SC15.** *P15 L14-15. These differences can be also a consequence of different game duration and format..*

  Thanks for your proposal. These contextual variables were included in the sentence.

- **R1_SC16.** *P15 L22-25. If reporting ranges, include first the lower value and then the higher (i.e. 7.6-9.9 and not 9.9-7.6). Furthermore, "y" after ref 46 should be removed.*

  Thanks for your suggestions. The values were ordered, and "y" was deleted.

- **R1_SC17.** *P15 L26. "Neuromuscular" should be replaced with "external".*

  Thanks for your proposal. "Neuromuscular" was changed by "external".

- **R1_SC18.** *P15 L25-27. Can these ranges be a consequence of the different playing position? Consider including this reasoning here.*

  Yes, we do agree with your comment. The ranges are consequence of the different playing position and the reason was added.

- **R1_SC19.** *P15 L31. "and which is most.." appears to be quite disconnected. Please rephrase and include reference.*

  Thanks for your comment. The sentence was rephrased to include the two approaches that is based on the previous inserted references.

- **R1_SC20.** *P16 L2-7. This sentence is too general; authors should include how to reach these results.*

  We really appreciate your suggestion. The sentence was rewritten including how to reach these results.

"Different investigations also use other PL^{TM}-dependent variables such as their segmentation by axes (PL_x, PL_y, PL_z) to analyze the specific contribution of each axis in the total workload on the technical-tactical skills or which axis is more related to fatigue during competition [42,47,49,85,91,92]. Otherwise, PL_{slow} quantifies the contribution of low-intensity workload (<2G) to the total workload of the players [44,79,87,93,94]. These two indexes allow higher accuracy and individualization of the demands performed by the athletes. The highest contribution to the external workload suffered by the athletes is from the vertical axis of movement, being over 50% of the total workload (y-axis > x-axis > z-axis). Also, the low-intensity workload represented between 35 and 50% of the cumulative PL. Therefore, the assessment of both indexes will be important for designing individualized technical-tactical-physical workloads and making possible the objective detection of players' deficiencies and optimum performance value enhancement".

- **R1_SC21.** *P16 L13. Accumulative or cumulative?*

  Thanks for your suggestion. The term "accumulative" was changed by "cumulative".

- **R1_SC22.** *P16 L25-27. This sentence does not appear grammatically correct. Please edit for clarity.*

  Thanks for your comment. The sentence was modified to appear grammatically correct.

- **R1_SC23.** *P17 L3-5. Please rephrase this sentence for clarity.*

  Thanks for your proposal. The sentence was rephrased.

- **R1_SC24.** *P17 L6-11.* This sentence should be rephrased as it is not fluid and clear.

  Yes, we do agree with your comment. The sentence was rephrased to improve the clarity and fluency.

- **R1_SC25.** *P17 L13-20. While I can understand the reasoning of authors here, the readers could be quite confused from these sentences. Please rephrase for clarity.*

  Thanks for your suggestion. The paragraph was rewritten to achieve a better clarity for the reader.

- **R1_SC26.** *P17 L23. Allows*

  Yes, we do agree with your comment. "Allow" was changed by "allows".

- **R1_SC27.** *P17 L26-27. This sentence is not clear enough. Are you referring that only 25.4% of studies reported BOTH validity and reliability?*

  We really appreciate your comment. 25.4% of studies reported both validity and reliability, 31.4% only the reliability, and 8.5% only the validity. The sentence was modified accordingly.

- **R1_SC28.** *P17 L27-30. Please rephrase this sentence as there are too many repetitions. Make this sentence clearer.*

  Thanks for your comment. The sentence was rewritten to delete too many repetitions and to improve the clarity.

- **R1_SC29.** *P18 L12. Maybe consider including "in different context and sports" to conclude this sentence.*

  Yes, we do agree with your suggestion. The terms "in different context and sports" have been added to conclude this sentence.

- **R1_SC30.** *P19 L14-15. I do not agree with this reasoning here. sRPE model is applied to quantify internal workload, which is a different variable than external workload. Why should we compare two different models measuring different variables? Please remove any comparison of accelerometers with sRPE model.*

  Thanks for your suggestion. The comparisons between RPE model and accelerometers have been removed.

- **R1_SC31.** *P19 L10. This comment refers to the entire manuscript. Consider a better use of RPE as in some occasions it has been used wrongly. Overall, RPE is not a load measurement; it is the rating of perceived exertion. Session RPE (sRPE) is the rating of perceived exertion for an entire training/competition session. sRPE workload is the workload quantified using the sRPE method. It is fundamental to use correct terms within a systematic review. See the following reference and edit within the manuscript accordingly. Reference: Impellizzeri FM, Marcora SM, Coutts AJ. Internal and External Training Load: 15 Years On. Int J Sports Physiol Perform. 2019 Feb 1;14(2):270-273. doi: 10.1123/ijspp.2018-0935. Epub 2019 Jan 6*

  Thanks for your suggestion. The use of RPE and sRPE were checked throughout the manuscript following the given reference.

- **R1_SC32.** *P19 L12-17. According to my previous comment (P4 L12-14) I do not fully agree with this statement as TMA in some cases can be more adequate for workload (and not "load" here - edit) quantification. Authors should consider differentiating manual than automatic and semi-automatic systems of TMA. Manual TMA can be used to identify static movements performed with high-intensity efforts (e.g. body contacts, low jumps) better than accelerometers (that do not measure low static movements without important accelerations), while I agree that automatic video-based analysis can underestimate workload. Edit this section accordingly.*

  Thanks for your suggestion. This section was edited accordingly.

<u>Reviewer 2</u>

General comments

- **R2_GC1.** *The reviewer thanks for the quick and solid revision of the submitted manuscript. The authors addressed all comments and did a great job in the implementation. Some of the new additions are very valuable.*
  There are few remaining concerns related to old and new parts of the manuscript that can easily be fixed. After such minor revision, the reviewer suggests that there is no further reviewing round required.

  Authors really appreciate all your comments provided throughout the peer-review process of the manuscript. All of them have contributed to improve the quality and clarity of the manuscript.

- **R2_GC2.** *First, there are issues with the pdf file received for revision that make revision difficult. The reviewer cannot tell if this is due to technical problems or caused by the authors. Therefore, addressing both authors and editor, the problems should be fixed in future submissions and reviewing processes. The problems are:*
  1. The pdf for revision does not include line numbers any longer.
  2. The changes highlighted in red to not show the original text that was removed (in contrast to the changes highlighted in blue; these changes were completely tracked).
  3. Some of the previous reviewer's comments are incorrectly presented. Examples are:
  3.1. R2_SC5. L52-52: The reviewer's comment was not "Third, "to explain" can be replaced by "to explain" as it was claimed. The reviewer's comment was "Third, "with the purpose of explaining" can be replaced by "to explain"."
  3.1. R2_SC8. L65: The reviewer's comment was not "Shorten: "to obtain" instead of "to obtain"". The reviewer's comment was "Shorten: "to obtain" instead of "with the aim of obtaining""
  Due to missing line numbers, the reviewer apologies to refer to chapters, paragraphs, and sentences.
  Second, to the authors: When responding to comments in such way "We really appreciate your suggestions. All of them have been considered to improve the final version of the manuscript", please inform where and how such considerations were implemented, especially for general comments that do not refer to specific lines.

  We really appreciate your comments. Firstly, authors apologize you due to not include the line numbers in the reviewed manuscript. The authors hope that you have been able to detect the changes made following the assigned codes and the red text. Following your suggestions, all changes have been made with tracked changes in this new revision round.

Specific comments

Abstract

- **R2_SC1.** *P2 L17. Correct is as it was suggested "A great number [...] makeS" because ONE number is the subject in this sentence.*

  Thanks for your comment. "Make" was changed by "makes".

Methods

- **R2_SC2.** *P4 L28. Sentence: Change "peer-reviewed manuscripts (scientific papers)" to "peer-reviewed, scientific papers" because, technically, a manuscript is not the same as a*

paper and not all peer-reviewed products are scientific products and vice-versa; plus, it is shorter.

Yes, we do agree with your comment. The terms were changed as you suggested.

- **R2_SC3.** *P5 L14. Paragraph: "genres" is assumed to be a typo. Change to "gender" or "sex" and be consistent in which one of the two is the appropriate to be used in this manuscript.*

  Thank you very much for your positive feedback. The term "sex" was used throughout the manuscript.

- **R2_SC4.** *P6 L13. Paragraph: The last sentence "In the present study, a value of 0.93 was obtained [...]" is a result of this study and should be moved to the results section. In the methods, only state that Kappa and 95% CI were calculated.*

  We really appreciate your suggestions and we agree with them. The last sentence was moved to results section. Besides, in the methods section, only state that Kappa and 95% CI were calculated.

Discussion

- **R2_SC5.** *P12 L21-22. Paragraph: 2. Sentence: Change to "Most studies [...] and DID not PROVIDE DISTINCT training [...]"*

  Thanks for your suggestion. The sentence was changed accordingly.

- **R2_SC6.** *P12 L26 Paragraph: Last sentence: Change "to possibility the comparisons between sports contexts" to "to allow for comparison between sports contexts"*

  Yes, we agree with your comment. The sentence was changed accordingly.

- **R2_SC7.** *P12 L27-28 Paragraph: 1. Sentence: The reviewer thinks it should be "in training than IN competition". Also, remove "but" and write "four OTHER articles".*

  Thanks for your proposal. The sentence was changed accordingly.

- **R2_SC8.** *P13 L1-2 Paragraph: 2. Sentence: It is clarified now, but the reviewer suggests rephrasing as follows: "[...] individualize the training sessions accounting for conditions (e.g. day [...]"*

  Thanks for your comment. The text was modified according to your proposal.

- **R2_SC9.** *P13 L6-10. Paragraph: Last sentence: Please make a full stop before and after Montgomery's findings. Then replace Ritchie's findings by: "Ritchie et al. [51] found greater workload in training compared with matches during the pre-season (PL: 19851985±745 vs. 1010±290) and the opposite during the in-season (PL: 1014±383 vs. 1320±195)."*

We really appreciate your suggestion. The sentence has been rewritten in the new version of the manuscript according to your proposal.

- **R2_SC10.** *P14 L8-9. Paragraph, new change: There is a wrong full stop after "[13,46]" and before "or by"; remove it. Change "Local Position Measurement (x/y axis)" to "horizontal Local Position Measurement (LPM)".*

  Yes, we do agree with your comment. The full stop was deleted. The word "by" was deleted. "Local Position Measurement (x/y axis)" was changed by "horizontal LPM (x/y axis)".

- **R2_SC11.** *P15 L10. Paragraph, middle: Change "are from" to "range from". What are these numbers used in soccer? If it was the number of occurrences, then write "the NUMBER OF impacts". If it was the actual impacts, use a unit.*

  Thanks for your suggestion. "are from" was changed by "range from" and the word "number of" was added before "impacts".

- **R2_SC12.** *P15 L22. Paragraph: There seems to be a mistake where, in the list of sports, one sport between soccer and netball is referred to as "y". It is the one backed-up by reference number 84.*

  Thanks for your appreciation. The word "y" was deleted.

- **R2_SC13.** *P16 L25. Paragraph, 2. Sentence: First, change to "Most studies". Second, the sentence is grammatically wrong, and its meaning is unclear. Consider: "Most studies showed that triaxial accelerometers use inertia sensors"*

  We really appreciate your suggestion. The sentence was rewritten completely.

  "Most studies with the purpose to detect movements patterns in invasion team sports through accelerometers presented a sampling frequency of 100 Hz".

- **R2_SC14.** *P17 L7-9. Paragraph, 3. Sentence: Change to "[...] detect three-dimensional movement and, consequently, to calculate the external workload index, which requires the acceleration in the three axes".*

  Thanks for your comment. The sentence was modified accordingly.

- **R2_SC15.** *P17 L9. Paragraph, 4. Sentence: Change to "[...] studies specified the number of accelerometers used in the devices."*

  Thanks for your comment. The sentence was modified accordingly

- **R2_SC16.** *P17 L9-11 Paragraph, 5. Sentence: Replace by "Moreover, the output range of each accelerometer is important and should be specified."*
- **R2_SC17.** *P17 L12. Sentence: it states "four accelerometers with different output ranges" but then two are identical (i.e. ±16g). Please correct by removing "different".*

  Thanks for your comment. The paragraph was rewritten to include the proposed changes.

“On the other hand, only 9 studies specified the number of accelerometers used in the devices. Moreover, the number of accelerometers that compose the device is not important unless the range output of each accelerometer is considered. WIMU PRO are composed by four accelerometers with specific output ranges ±16g, ±16g, ±32g and ± 400g [100,104,105] while Optimeye S5 are composed by three ±16g accelerometers [106]”.

- **R2_SC18.** *P17 L31-32 to P18 L1-7. Paragraph: That is indeed worrying. Please add one short clarification if studies, that did not report validity and reliability, did refer to literature reference for this purpose instead (yes/no?). In the subsequent paragraph you state that some studies did refer to inappropriate investigations. That means some do use references. So, simply state how many did (in percentage of the reviewed studies). That is enough.*

We really appreciate your suggestion. The aspects mentioned have been clarified in the new version of the manuscript. Besides, the percentage of studies was inserted as you recommended.

“From the different studies that cited the reliability and validity of accelerometers, it has also been found that 15 investigations (12.7%) cited the reliability and validity of other devices that are different from those used in their research. Investigations measured with Optimeye and Team 2.5 devices (Catapult Sports) [42,54,62,87,110–114], ZXY Sportracking (Radionor Communications) [26], X8-mini (Gulfcoast Data Concept) [24,25,29], Actilife v12 (ActiGraph) [115], and Viper V2 (StatSport) [116] cited the validity study of Barrett et al. [19] and reliability of Boyd et al. [16] realized with MinimaxX devices (Catapult Sports). Finally, 34.7% of studies not reported validity or reliability and did not refer to literature citations for this purpose”.
