# Supplementary material for: Accelerometry as a method for external workload monitoring in invasion team sports. A systematic review
Source: PLoS One. 2020 Aug 25;15(8):e0236643. doi: 10.1371/journal.pone.0236643 (PMC7447012; doi:10.1371/journal.pone.0236643)
Supplement: Attachment — Submitted filename: Response to Reviewers.docx [file pone.0236643.s009.docx]

Dear Editor,

We have carefully considered all reviewers' considerations of the paper (PONE-D-19-33961_R2). Please find enclosed our detailed answers to reviewers' queries. The authors declare that the manuscript is original and has not been considered for publication elsewhere. Additionally, the authors had approved the paper for release and agree with its content.

**Reviewer 1**

**General comments**

- **R1_GC1.** *The authors did a big effort editing the manuscript during the revision process. The manuscript has been greatly improved and will be a valuable addition to the literature. In my opinion, the manuscript should be considered acceptable for publication. I would recommend a final check for grammar, spelling, and punctuation mistakes during the editorial editing/formatting process (e.g. page 3, line 5 “suggestS”).*

The authors really appreciate all your kindly comments related to the previous peer-review step of our manuscript. Besides, the authors thanks the effort realized by the reviewer throughout the peer-review process of the manuscript to improve the clarity and quality of the manuscript overall. Finally, a final check for grammar, spelling, and punctuation mistakes was performed following the reviewer's suggestions.

**Reviewer 2**

**General comments**

- **R2_GC1.** *The The authors addressed all comments and fixed most issues. Some new mistakes emerged in the adaptations. For all cases, the reviewer provides concrete solutions. In the specific comments, the reviewer refers to page and line numbers of the revised manuscript showing the tracked changes. The reviewer suggests acceptance of the manuscript after the implementation of the provided solutions.*

The authors want to thank the effort realized by the reviewer throughout the peer-review process of the manuscript. All of the proposed changes have contributed to improving the quality and clarity of the manuscript. Finally, the authors have incorporated the different suggestions proposed in the last revision with the implementation of the provided solutions.

**Specific comments**

***Methods***

- **R2_SC1.** *P5, L2: Change “manuscript” to “paper” as the currently submitted piece of work is a manuscript (L1) that reviews published papers. This is how terms are correct. When published, a manuscript is a paper.*

Thanks for your comment. The term “manuscript” was changed by “paper”.

***Discussion***

- **R2_SC2.** *P12, L22: A new error appeared after revision: Correct is “did not provide” (not: provideD)*

Yes, we do agree with your comment. The term was changed as you suggested.

- **R2_SC3.** *P14, L3-4: The meaning of the new adaptation is unclear to me (studies are no consequence of different schedules!). The sentence is also grammatically incorrect. The reviewer can just assume that the authors meant something like: “Higher competition workload reported in some studies may be the consequence of differences in weekly schedules, not accounting for conditions […]” If this message is in line with the authors’ intentions, then I recommend to use this phrase.*

Thank you very much for your positive feedback. The sentence was rephrased following your considerations.

- **R2_SC4.** *P13, L8+9: 1. In both brackets you need to write “(PL: [numbers])”, otherwise no one knows what these numbers are. 2. You may change “in-season” to “competitive season”.*

We really appreciate your suggestions and we agree with them. The word PL was added before the numbers into the brackets and “in-season” was changed by “competitive season”.

- **R2_SC5.** *P14, L8+9: Why was “Global Navigation Satellite Systems” and “Local Position Measurement” replaced by short terms? Check if the short terms have been introduced previously, otherwise introduce them here*

Thanks for your suggestion. The terms “Global Navigation Satellite Systems, GNSS” and “Local Position Measurement, LPM” have been introduced in the introduction section (Page 3, L17-18). In this sense, reviewer 1 suggest replaced them by short terms in the last revision.

- **R2_SC6.** *P14, L9: Remove “(x/y axis)” because, first, this is redundant and, second, horizontal is the better description anyway because x/y is a matter of definition and could be anything (therefore, would require a definition).*

Yes, we agree with your comment. The term (x/y axis) was deleted.

- **R2_SC7.** *P15, L8-10: Previous suggestion was misunderstood, and adaptations are not correct anymore (the threshold does not range!). Change to full stop after the rugby and American football sentence (L16). Then, “In soccer, the detection threshold is 5G, and the number of impacts range from 490±310 to 613±329 [83].”*

Thanks for your proposal. The authors apologize to you for the mistake committed. The sentence was rephrased as you recommended.

- **R2_SC8.** *P16, L27-28: Change LESS sampling rate and accuracy to LOWER sampling rate and accuracy.*

Thanks for your comment. The text was modified according to your proposal.

- **R2_SC9.** *P17, L9-10: Remove “Moreover” and “that compose the device” and change to “The number of accelerometers is only important if the output range […]. Do not mess up the order in output range/range output.*

We really appreciate your suggestion. The sentence has been rewritten in the new version of the manuscript according to your proposal. Throughout the manuscript output range was used as you suggested.

- **R2_SC10.** *P17, L16: Here is also incorrectly written “range output”. Change to “output range”.*

Yes, we do agree with your comment. Throughout the manuscript output range was used as you suggested.

- **R2_SC11.** *P17, L30-32: Change to “Among studies that cited the reliability and validity of accelerometers, 15 investigations (i.e. 12.7%) cited the reliability and validity of other devices that were not used in the respective research.”*

Thanks for your suggestion. The sentence was rephrased as you suggested.

- **R2_SC12.** *P19, L28-29: Change to “NOTEWORTHY, 34.7% of THE studies DID not report [not: reportED!] THE validity or reliability and did also not refer to literature FINDINGS for this purpose.”*

Thanks for your appreciation. The sentence was changed following your recommendations.
